# Self-organized criticality in a mesoscopic model of excitatory-inhibitory neuronal populations by short-term and long-term synaptic plasticity

**DOI:** 10.3389/fncom.2022.910735

**Published:** 2022-10-10

**Authors:** Masud Ehsani, Jürgen Jost

**Affiliations:** ^1^Max Planck Institute for Mathematics in Sciences, Leipzig, Germany; ^2^Santa Fe Institute, Santa Fe, NM, United States

**Keywords:** critical brain hypothesis, Bogdanov-Takens bifurcation, scale-free avalanches, self-organization, STDP, short term depression

## Abstract

Dynamics of an interconnected population of excitatory and inhibitory spiking neurons wandering around a Bogdanov-Takens (BT) bifurcation point can generate the observed scale-free avalanches at the population level and the highly variable spike patterns of individual neurons. These characteristics match experimental findings for spontaneous intrinsic activity in the brain. In this paper, we address the mechanisms causing the system to get and remain near this BT point. We propose an effective stochastic neural field model which captures the dynamics of the mean-field model. We show how the network tunes itself through local long-term synaptic plasticity by STDP and short-term synaptic depression to be close to this bifurcation point. The mesoscopic model that we derive matches the directed percolation model at the absorbing state phase transition.

## 1. Introduction

Neural networks as complex dynamical systems with many degrees of freedom varying over different time scales can be seen as a self-tuning system that attains a dynamical regime where the system can carry out its task. On the other hand, spontaneous intrinsic activity of cortical neural assemblies in absence of any information processing task can be perceived as a substrate for the neural dynamics which can give us insights into the preferred dynamical regime and the goal of self-organization processes. Dynamic and functional characteristics of spontaneous activity are connected to the structural architecture of the brain as well as the ongoing self-organization process. Experimental findings on different temporal and spatial resolutions highlight the scale-free characteristic of spontaneous activity. When recorded by coarse-grained methods like EEG and MEG, spontaneous brain activity shows nested oscillations with a power spectrum that indicates scale-free properties, i.e., *P*(*f*) ∝ 1/*f*^β^ (Linkenkaer-Hansen et al., 2001; Miller et al., 2009; Hardstone et al., 2012). Microelectrode recordings of smaller cortical regions show activity in the form of avalanches with power-law distributions of size and duration in different setups such as cultured slices of rat cortex (Beggs and Plenz, 2003), awake monkeys (Petermann et al., 2009), in cerebral cortex and hippocampus of anesthetized, asleep, and awake rats (Ribeiro et al., 2010), and the visual cortex of an anesthetized cat (Hahn et al., 2010).

One explanation for the scale-free characteristics is that cortical networks operate near a critical second-order phase transition (Chialvo, 2004; Tagliazucchi and Chialvo, 2013). On one hand, close to the edge of an active-inactive phase transition, local populations of neurons in the cortex would be in an idle state ready for processing information, but at the same time away from overactivation. On the other hand, close to the phase transition, an order parameter, a macroscopic state different from the inactive state comes into existence. The emergence of a macroscopic mode of activity acts as the coordinator of individual neurons which are enumerated in quantity and prone to various kinds of noise to produce a cooperative large-scale activity (Chialvo, 2010).

The type of the phase transition and the dynamical regime that produces the aforementioned characteristics of avalanches have been studied in different neuronal models. Active-inactive phase transition in a purely excitatory population of neurons (Levina et al., 2007, 2009) and synchronization phase transition in an excitatory population coupled with short-term depression of synaptic resources (di Santo et al., 2018) have been proposed as the origin of avalanche dynamics. The main hypothesis is that system resides near the bifurcation point of the quiescent state. In an Inh.-Exc. network, Benayoun et al. proposed a stochastic model of spiking neurons which matches the Wilson-Cowan mean field in the limit of infinite system size that shows scale-free avalanches in the balanced state in which the sum of excitation and inhibition is much larger than the net difference between them (Benayoun et al., 2010). Under symmetry conditions on weights, the Jacobian has negative eigenvalues close to zero in the balanced state. de Candia et al. (2021) showed that this stochastic model exhibits second-order phase transition with scale-free avalanches and the interaction of noise and nonlinearity is the origin of this behavior. In this model, the Poisson firing of the neurons is preassumed and symmetric synaptic connections and *O*(*N*^−1^) scaling of weights is required for applying the linear noise approximation. Furthermore, the origin of the scale-free behavior and the bifurcation diagram of the model in a wider regime of parameters has not been studied. To investigate further the emergence of avalanches in the EI network, in Ehsani and Jost (2022), we used a bottom-up approach to obtain a single neuron's gain function subjected to fluctuating conductance-based synaptic currents and studied the linear Poisson firing regime for EI homogenous sparse population using Fokker-Planck formalism. We showed that the Bogdanov-Takens bifurcation point of the mean-field equations for dynamics of a sparse homogenous excitatory and inhibitory population of spiking neurons is the operating point of the system producing the characteristic spontaneous activity in the form of scale-free avalanches and Poisson firing of neurons. In this regime, the system is close to both the saddle-node bifurcation point at the low firing rate regime and Hopf-bifurcation of the quiescent fixed point. Tight temporal balance of inhibition and excitation at this state and Poisson firing of neurons is the origin of critical behavior. By mapping single population dynamics to a branching process, we have explained the emergence of power law exponents in the spiking neural network which coincides with critical exponents of the branching process.

To tune the system at the critical point, many modeling approaches and adaptive mechanisms have been suggested during the decades of research on the critical brain hypothesis. The self-organizing principle is mainly based on the model of neuronal dynamics and the choice of the control parameter. In the excitatory neuronal population, a SOC model that attracted much attention is introduced by Levina et al. (2007, 2009). In their model, short-term depression of excitatory synapses coupled with internal neuronal dynamics self-tunes the system at the edge of the active-inactive phase transition. In addition to self-organization by short-term depression in synapses which is also used in Peng and Beggs (2013) and di Santo et al. (2018), self-organization by other control parameters like degree of connectivity or synaptic strength has been studied (Bornholdt and Roehl, 2003; Rybarsch and Bornholdt, 2014). In Brochini et al. (2016), self-organization in stochastic spiking neuron model by short-term plasticity of the gain function instead of synaptic weights is introduced. Meisel and Gross (2009) introduced a self-organizing excitatory neural network by STDP. Scarpetta and Candia (2013) studied transient replay of stored patterns by STDP in a balanced network at a macroscopic phase transition induced by noise and also observed scale free avalanches and studied their statistics near the noise induced phase transition (Scarpetta et al., 2013).

Here, we consider the self-tuning of the system at the BT critical point of the EI interconnected network. The self-organizing parameter in our network is the balance of opposing forces resulting from the activities of inhibitory and excitatory populations, and the self-organizing mechanisms are long-term synaptic plasticity through the mechanism of Spike Timing Dependent Plasticity (STDP) and homeostatic short-term depression of the synapses. The former tunes the overall strength of excitatory and inhibitory pathways to be close to a balanced regime of these currents and the latter, which is based on the finite amount of resources in brain areas, acts as an adaptive mechanism that tunes micro populations of neurons subjected to fluctuating external inputs to attain the balance in a wider range of external input strengths. The importance of both types of synaptic plasticity in the emergence and tunning of the critical state has been also mentioned in a review article by Zeraati et al. (2021). Here, we show this phenomenon in the context of EI networks with long-term synaptic plasticity in all types of synapses which has not been studied previously.

For analytical analysis of STDP on average weight connections, we use the inhomogenous Poisson neuron assumption that has been studied in Kistler and van Hemmen (2000) and Burkitt et al. (2007). Under general conditions on inhibitory and excitatory STDP kernels, i.e., negative integral of the excitatory and positive integral of the inhibitory STDP kernels, learning results in a balanced internal state. This condition on kernels leads to stabilization of rates as also discussed in Kempter et al. (2001). We use the model of Tsodyks and Markram (1997) for short-term depression of the excitatory synapses which has been studied vastly (refer to for example Kistler and van Hemmen, 1999).

Using the Poisson firing assumption, we propose a microscopic Markovian model which captures the internal fluctuations in the network due to the finite size and matches the macroscopic mean-field equation by coarse-graining. Near the critical point, a phenomenological mesoscopic model for excitatory and inhibitory fields of activity is possible due to the time scale separation of slowly changing variables and fast degrees of freedom. We will show that the mesoscopic model corresponding to the neural field model near the local Bogdanov-Takens bifurcation point matches the Langevin description of the directed percolation process.

## 2. Materials and methods

### 2.1. Neuron model

We use an integrate and fire neuron model in which the change in the membrane voltage of the neuron receiving time dependent synaptic current *i*(*t*) follows


(1) $$\begin{array}{l} C\frac{dv\left(t\right)}{dt}=g_{Leak}\left(v_{Leak}-v\left(t\right)\right)+i\left(t\right) \end{array}$$

for *v*(*t*) < *v*_*th*_. When the membrane voltage reaches *v*_*th*_ = −50*mv*, the neuron spikes and immediately its membrane voltage resets to *v*_*rest*_ which is equal to *v*_*Leak*_ = −65*mv*.

In the following, we want to concentrate on a model with just one type of inhibitory and one type of excitatory synapses, which can be seen as the average effect of the two types of synapses. We can write the synaptic inhibitory and excitatory current as


(2) $$\begin{array}{l} \begin{array}{c} i\left(t\right)=g_{inh}\left(t\right)*\left(V_{Rinh}-v\left(t\right)\right)+g_{exc}\left(t\right)*\left(V_{Rexc}-v\left(t\right)\right) \end{array} \end{array}$$

*V*_*Rinh*_ and *V*_*Rexc*_ are the reverse potentials of excitatory and inhibitory ion channels and based on experimental studies we choose values of −80 and 0*mv* for them respectively. *g*_*Inh*_(*t*) and *g*_*Exc*_(*t*) are the conductances of inhibitory and excitatory ion channels. These conductances are changed by the inhibitory and excitatory input to the cell. Each spike of a presynaptic inhibitory or excitatory neuron *j* to a postsynaptic neuron *k* that is received by *k* at time *t*_0_ will change the inhibitory or excitatory ion channel conductance of the postsynaptic neuron for *t* > *t*_0_ according to:


(3) $$\begin{array}{l} g_{Inh}^{k}\left(t\right)=w_{kj}*g_{0}^{inh}*exp\left(-\frac{t-t_{0}}{\tau_{syn}^{inh}}\right)\\ g_{Exc}^{k}\left(t\right)=w_{kj}*g_{0}^{exc}*exp\left(-\frac{t-t_{0}}{\tau_{syn}^{exc}}\right) \end{array}$$

Here, we assume that the rise time of synaptic conductances is very small compared to other time scales in the model, and therefore, we modeled the synaptic current by a decay term with synaptic decay time constant τ_*syn*_ which we assume to be the same value of 5*ms* for both inhibitory and excitatory synapses.

### 2.2. Network architecture

In the remainder of this study, in the simulation, we consider a population of $N_{Exc}=2\;*\;10^{4}$ and *N*_*Inh*_ = 0.25 * *N*_*Exc*_ inhibitory spiking neurons with conductance-based currents introduced in this section. Each excitatory neuron in the population is randomly connected to $k_{EE}=\frac{N_{Exc}}{100}=200$ excitatory and $k_{EI}=\frac{k_{EE}}{4}$ inhibitory neurons and each inhibitory neuron is connected to *k*_*IE*_ = *k*_*EE*_ and $k_{II}=\frac{k_{EE}}{4}$ excitatory and inhibitory neurons, respectively. The weights of excitatory synaptic connections are in a range that 10 − 20 synchronous excitatory spikes suffice to depolarize the target neuron to the level of its firing threshold when it is initially at rest at the time of input arrival. Weights are being drawn from a log-normal probability density with low variance. Therefore, approximately $O\left(\sqrt{k_{EE}}\right)$ spikes are adequate for firing. Assuming homogeneity in the population as we have discussed in the introduction we can build a mean-field equation for the excitatory and inhibitory population in this sparse network, assuming each neuron receives input with the same statistics.

### 2.3. Avalanche regime of activity as desired operating point of the system

In Ehsani and Jost (2022), we have investigated the dynamics of excitatory and inhibitory (EI) sparsely connected populations of spiking leaky integrate neurons with conductance-based synapses. We have seen that close to the Bogdanov-Takens bifurcation point of the mean field equation, the output firing of the population is in the form of avalanches with scale free size and duration distribution. This matches the characteristics of low firing spontaneous activity in the cortex. By linearizing gain functions and excitatory and inhibitory nullclines, we approximated the location of the BT bifurcation point. This point in the control parameter phase space corresponds to the internal balance of excitation and inhibition and a slight excess of external excitatory input to the excitatory population. Due to the tight balance of average excitation and inhibition currents, the firing of the individual cells is fluctuation-driven. Around the BT point, the spiking of neurons is a Poisson process and the population average membrane potential of neurons is approximately at the middle of the operating interval [*V*_*rest*_, *V*_*th*_]. Moreover, the EI network is close to both oscillatory and active-inactive phase transition regimes.

At equilibrium, population rates satisfy a system of fixed point equations of the form:


(4) $$\begin{array}{l} \rho_{I}=g^{I}\left(\rho_{I},c_{EI}\rho_{E}+c_{II}\rho_{I}+d\rho_{Ext}^{I}\right)-z_{0}\\ \rho_{E}=g^{E}\left(\rho_{I},c_{EE}\rho_{E}+c_{EI}\rho_{I}+d\rho_{Ext}^{E}\right)-z_{0} \end{array}$$

where *c*_*xy*_ = *ck*_*xy*_*w*_*xy*_(*V*_*R*_*y*__ − 〈*V*_*x*_〉). *k*_*xy*_ is the average number of synaptic connections between neurons of population *y* to neurons in population *x* with an average strength of *w*_*xy*_. *z*_0_ is a constant that depends on *V*_*rest*_, *V*_*th*_, the maximal rates and the SD of the input. *V*_*R*_*y*__ is the reverse potential level of a neuron of type *y* and 〈*V*_*x*_〉 is the average potential level of neurons in population *x* that can be written in fluctuation driven the firing regime as:


(5) $$\begin{array}{l} <V_{x}>=\frac{g_{L}V_{L}+g_{exc}^{0}w_{xE}\rho_{E}\tau V_{Rexc}+g_{inh}^{0}w_{xI}\rho_{I}\tau V_{Rinh}}{g_{L}+g_{exc}^{0}w_{xE}\rho_{E}\tau +g_{inh}^{0}w_{xI}\rho_{I}\tau } \end{array}$$

τ is the synaptic current decay time constant, *g*_*L*_, *g*_*exc*_ and *g*_*inh*_ are the baseline conductances of leaky, excitatory and inhibitory ion gates, respectively. We took *w*_*EE*_ and $\rho_{Ext}^{E}$ as control parameters and analyzed solutions to Equation (4). By substituting nonlinear gain functions with their corresponding linearization in the Poisson firing regime, we showed that the low BT point is located close to the matching condition for the *y*-intercept and the slopes of the linearized nullclines, which are written as:


(6) $$\begin{array}{l} c_{EE}^{*}=\frac{c_{EI}c_{EI}}{c_{II}}\\ \rho_{Ext}^{E^{*}}=\frac{c_{EE}^{*}}{c_{IE}}\left(\rho_{Ext}^{I}-d\right)+d \end{array}$$

where *d* is a constant equal to $\frac{g_{L}\left(V_{rest}-V_{th}\right)}{\tau *g_{exc}^{0}*\left(V_{th}-V_{R_{exc}}\right)}$.

**Figure 1:**
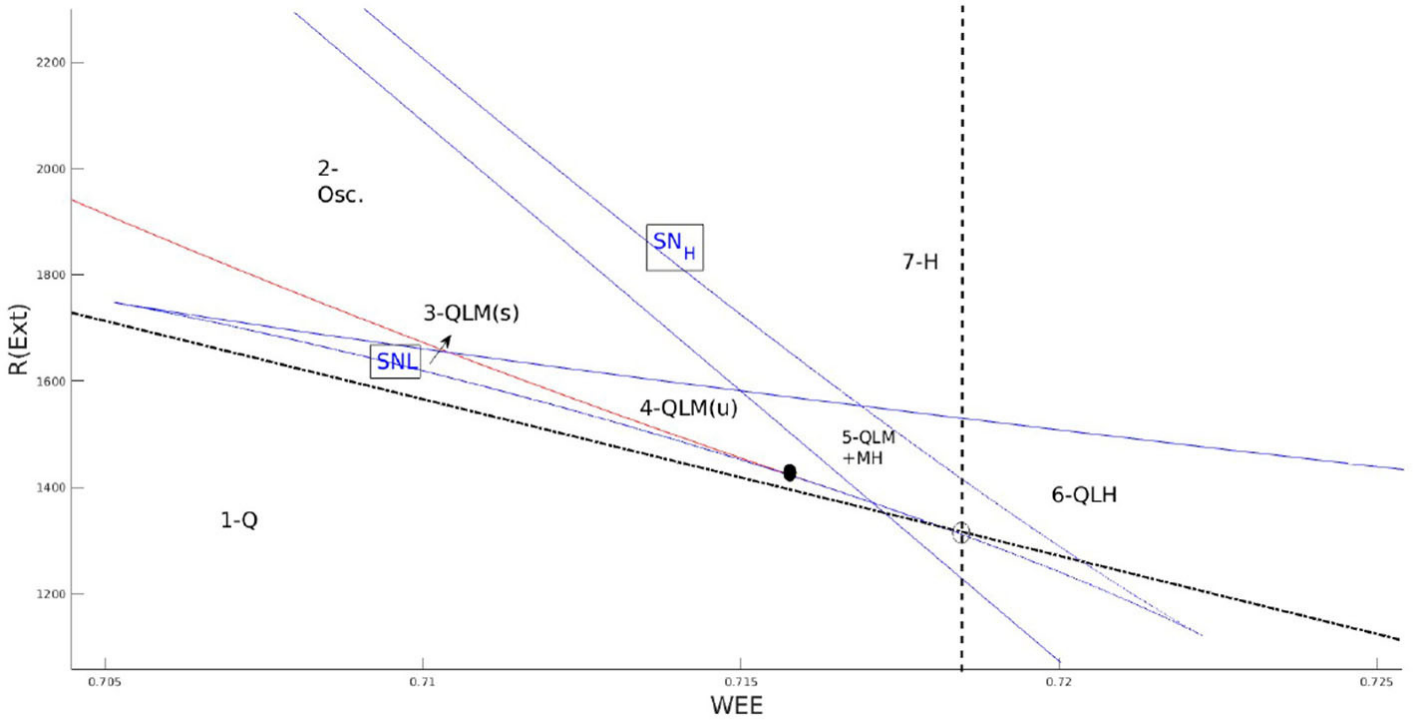
Zoom in on the local bifurcation diagram at low firing rates and the corresponding regimes of phase space with different numbers of fixed points. The dashed line is the condition on the equal slope of linearized nullclines and the semi-dashed line is the condition on equal y-intercepts. BT point (black dot) is close to the intersection of these lines. In the labeling of regions (Q) denotes quiescent state fixed point, (L) is the fixed point in low firing rate, (M) is the fixed point in the linear section, and (H) is the high firing fixed point.

**Figure 2:**
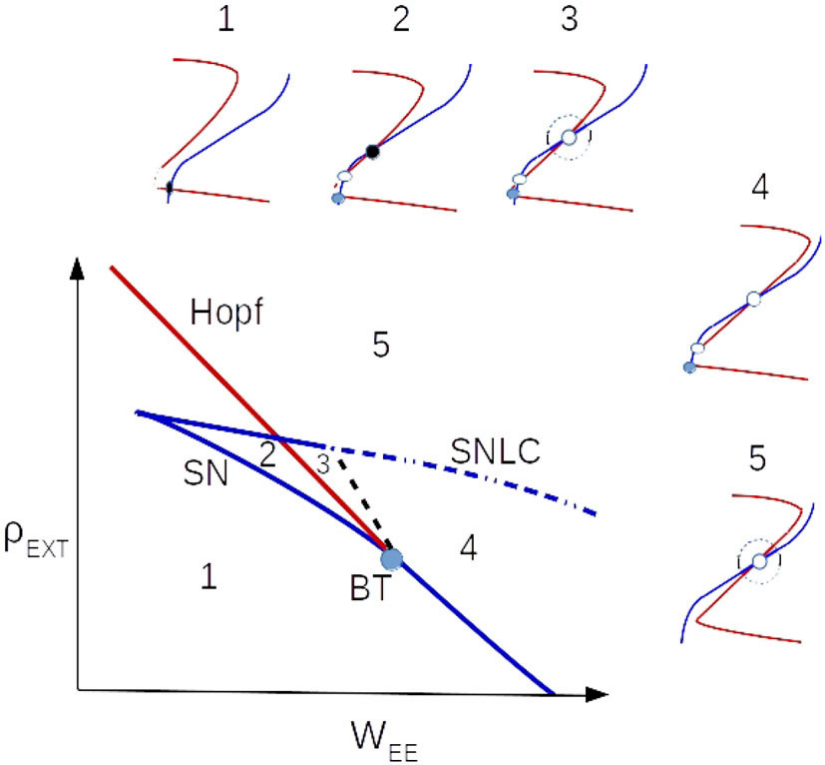
Nullclines' arrangements near the BT point in the local bifurcation diagram. The black dashed line is saddle-separatrix loop bifurcation and the blue dotted-dashed is saddle-node on limit cycle (SNLC) bifurcation line.

Figure 1 shows the location of the BT point on the local bifurcation diagram and matching condition of Equation 6. Nullclines of Equation 4 near the BT point are depicted in Figure 2. Close to the BT point, the volume of the basin of attraction of the quiescent state is small, and internal noise can make the system escape from it. Activity grows and decays back to the quiescent state along heteroclinic orbits connecting the two saddle points in the low firing rate regime which coincides with the slow manifold of the fixed points. Along this slow manifold, there is a tight temporal balance of excitation and inhibition in the forms of avalanches of highly variable sizes. This results in balanced currents at each cell leading to Poisson firing of individual neurons. At the population level, the temporal balance of excitation and inhibition cancels out the average current to the cells while the fluctuation in the current which is proportional to the average rates leads to a branching factor close to unity for both excitatory and inhibitory populations. The mapping to the branching process on a single EI network explains the emergence of power law exponents in the spiking neural network which agrees with the mean field branching process.

### 2.4. Synaptic plasticity

We will analyze and simulate a network in which neurons will adapt their connections according to the Spike-timing dependent plasticity (STDP) paradigm (Gerstner et al., 1996), which provides a foundation for temporal coding.

In STDP, the weight of a connection is modified depending on the time interval between pairs of pre- and post-synaptic spikes. For every pair, the weight of the synapse changes according to the equations


(7) $$\begin{array}{l} \Delta w\left(\Delta t\right)=\{\begin{array}{cc} f_{+}\left(w\right)K_{+}\left(\Delta t\right) & \text{if }\Delta t\geq 0\\ -f_{-}\left(w\right)K_{-}\left(\Delta t\right) & \text{if }\Delta t<0 \end{array} \end{array}$$

where Δ*t* = *t*_*post*_ − *t*_*pre*_ is the time difference between the postsynaptic spike and the presynaptic one. The functions *f*_+_ and *f*_−_ model the dependence of the weight change on the current value of the synaptic weights. *K*_+_ and *K*_−_, called STDP kernels, usually are decaying functions of time which reflects the fact that closer pre- and post-synaptic spikes generate stronger weight changes. Usually, we model the kernels by a single exponential such as $K_{+}=A_{+}e^{-\frac{\left|\Delta t\right|}{\tau_{s_{+}}}}$ and $K_{-}=A_{-}e^{-\frac{\left|\Delta t\right|}{\tau_{s_{-}}}}$. As it is evident from equation (7) when the postsynaptic neuron fires after the presynaptic neuron, the strength of the connection increases and it decreases for the opposite temporal order. We assume the same type of the STDP rule for both inhibitory and excitatory connections although with different kernels. In the following, we suppose that the dependence of STDP on the synaptic weight is negligible and therefore replace the functions *f*_+_ and *f*_−_ by a constant which is then absorbed into the kernels. In this case, we have to assume a saturation level for the maximum strength of the synapses, $w_{max}^{E}$ and $w_{max}^{I}$.

We use the model of Tsodyks and Markram (1997) for short-term depression of the excitatory synapses reduces the outgoing synaptic efficacy of excitatory synapses to an excitatory neuron in case of a high rate of presynaptic activity. To model the STP effect, we assume that the effective utility of the excitatory synapses of neuron *j* to the other neurons is proportional to the fraction of the available synaptic resources *u*. The decrease of neurotransmitters at the synapses and depression in release probability due to consecutive uses of neurotransmitters in previous spikes of the presynaptic neuron are the sources of STP. We assume by each spike of a presynaptic neuron, *u* is reduced by the factor *qu* and then recovers with the time constant τ_*STP*_ which is of order 100*ms* to a few seconds. Therefore, synaptic efficacy of the postsynaptic synapse of neuron *j* evolves as:


(8) $$\begin{array}{l} \frac{du_{j}}{dt}=\frac{1}{\tau_{STP}}\left(1-u_{j}\right)-qu_{j}\underset{k}{\sum }\delta \left(t-t_{k}^{j}\right) \end{array}$$

## 3. Results

### 3.1. Long term synaptic plasticity by STDP tunes synaptic weights close to the balanced state

A typical neuron in the cortex has 10^3^ − 10^4^ synaptic connections with 80% of them of excitatory type and 20% of inhibitory type. On the other hand, even in the resting state, neurons on average have a non-zero firing rate with an average rate of 1*Hz* and their spike trains are very noisy with exponential inter-spike interval distribution indicating that the spiking of individual neurons is a Poisson point process. Yet another experimental fact about synaptic strength between neuron states is that usually, 10 − 20 presynaptic synchronous spikes suffice to bring a typical neuron to the firing threshold. If we take τ_*m*_ = 20*ms* as the membrane potential decay time constant, then during this time window a typical neuron receives 20 − 200 excitatory spikes, which are enough for the neuron to periodically spike at a very high rate. To avoid this, the inhibitory input in this time window should largely cancel the excitatory current. Therefore, for the average currents to maintain the average membrane potential below the threshold in order to avoid a high firing state and produce high variability in the spike trains, inhibitory and excitatory currents should be balanced. Dynamical balance of excitation and inhibition ensures a low level of activity, i.e., an asynchronous firing state. In the following, we present a synaptic plasticity rule which tunes the average synaptic weights to the balanced state.

We derive an equation for the evolution of the average and the variance of weights between excitatory and inhibitory neurons during the plasticity period.

Spike-timing dependent plasticity (Equation 7) changes synaptic weights on a very slow time scale compared to firing dynamics of the neurons, therefore, during a time period of [*t, t* + Δ*t*] where Δ*t* is long in comparison with the inter-spike time interval but small enough that the change in the weight *w*_*ij*_ of the synapse from neuron *j* to neuron *i* is infinitesimal, we can write:


(9) $$\begin{array}{l} \Delta w_{ij}=\int_{t}^{t+\Delta t}\int_{0}^{\infty }S_{j}\left(s\right)S_{i}\left(s+\delta \right)f_{+}\left(w_{ij}\right)K_{+}\left(\delta \right)d\delta ds\\ \;+\int_{t}^{t+\Delta t}\int_{0}^{\infty }S_{i}\left(s\right)S_{j}\left(s-\delta \right)f_{-}\left(w_{ij}\right)K_{-}\left(\delta \right)d\delta ds \end{array}$$

where *S*_*i*_(*t*) and *S*_*j*_(*t*) are the spike trains of the presynaptic and the postsynaptic neurons. Assuming that during this period, the firing rate of the output neuron is constant on average and there exist many pre- and post-synaptic spikes, we can write the mean change in the incoming synaptic weights to the neuron *i* as,


(10) $$\begin{array}{l} \frac{{\langle \Delta w_{ij}\rangle }_{j}}{\Delta T}=\int_{0}^{\infty }{\langle S_{j}\left(s\right)S_{i}\left(s+\delta \right)\rangle }_{j}K_{+}\left(\delta \right)d\delta -\int_{0}^{\infty }{\langle S_{j}\left(s\right)S_{i}\left(s-\delta \right)\rangle }_{j}K_{-}\left(\delta \right)d\delta \end{array}$$

We want to investigate the evolution of the synaptic weights in the EI population in an asynchronous irregular state. Therefore, we assume that in the regime of spontaneous activity, neurons are firing as a Poisson process. Moreover, to estimate the cross-correlation of the pre- and the post-synaptic spike train we argue that the excitatory input to the cell has a positive correlation with preceding spikes in the target neuron. The magnitude of this excess correlation depends on the weight of the synapse and it is restricted to the time window before the firing of the postsynaptic neurons. With this in mind, we use the following approximation introduced in van Rossum et al. (2000) and Câteau and Fukai (2003) for the cross-correlations of spike trains to account for the causal contributions of presynaptic spikes to the postsynaptic ones of a synapse with the strength *w*_*i*_:


(11) $$\begin{array}{l} \langle S_{pre}^{E}\left(s\right)S_{post}^{E}\left(s+\delta \right)\rangle =\rho_{pre}^{E}\rho_{post}+\rho_{pre}^{E}w_{i}\left(V_{Rexc}-V_{i}\right)\gamma^{E}\left(\delta \right)\\ \langle S_{pre}^{I}\left(s\right)S_{post}^{E}\left(s+\delta \right)\rangle =\rho_{pre}^{I}\rho_{post}-\rho_{pre}^{I}w_{i}\left(V_{i}-V_{Rinh}\right)\gamma^{I}\left(\delta \right) \end{array}$$

Here, *V*_*i*_ is the voltage level of the postsynaptic neuron. As the second terms in both equations encode the excess correlation (anticorrelation) of the presynaptic excitatory(inhibitory) input preceding the firing at the postsynaptic neuron, we set γ^*I*^(δ) = γ^*E*^(δ) = 0 for δ < 0. For positive values of δ, this function which is independent of the rates and the weights of the synapses encodes the causal effect of the presynaptic spike which arrives δ units of time before firing of the postsynaptic neuron. Therefore, it is a decaying function of δ. Moreover, we have assumed the dependence on the weight of the synapse to be of a linear form, which is a good approximation in the regime of small synaptic weights. Inserting the above approximation and labeling the STDP kernels of Exc. to Exc. (EE) and Exc. to Inh. (IE) synapses as *K*^*E*^ and the STDP kernels of Inh. to Inh. (II) and Inh. to Exc. (EI) synapses as *K*^*I*^, we can write the evolution of the average excitatory and inhibitory synaptic strength to the neuron *i* as


(12) $$\begin{array}{l} \frac{d\langle w_{ij}^{E}\rangle }{dt}=\langle \rho_{j}^{E}\rangle \rho_{i}\left({\bar{K}}_{+}^{E}-{\bar{K}}_{-}^{E}\right)+\langle \rho_{j}^{E}\rangle \langle w_{ij}^{E}\rangle \left(V_{Rexc}-\langle V_{i}\rangle \right)\bar{K_{+}^{E}\gamma^{E}}\\ \frac{d\langle w_{ik}^{I}\rangle }{dt}=\langle \rho_{k}^{I}\rangle \rho_{i}\left({\bar{K}}_{+}^{I}-{\bar{K}}_{-}^{I}\right)-\langle \rho_{k}^{I}\rangle \langle w_{ik}^{I}\rangle \left(\langle V_{i}\rangle -V_{Rinh}\right)\bar{K_{+}^{I}\gamma^{I}} \end{array}$$

Here, bars denote integrals of the kernels on the positive or negative real lines. In the population of sparsely connected and sufficiently homogeneous neurons, in terms of the number of connections of each neuron, and the regime of asynchronous homogeneous firing, i.e., when all the neurons fire with the same average rate but with a random phase of firing between them, the average weights evolve as


(13) $$\begin{array}{l} \frac{dw_{EE}}{dt}=\rho_{E}^{2}\hat{K^{E}}+\rho_{E}w_{EE}\left(V_{Rexc}-\langle V^{E}\rangle \right)\bar{K_{+}^{E}\gamma^{E}}\\ \frac{dw_{EI}}{dt}=\rho_{E}\rho_{I}\hat{K^{I}}-\rho_{I}w_{EI}\left(\langle V^{E}\rangle -V_{Rinh}\right)\bar{K_{+}^{I}\gamma^{I}}\\ \frac{dw_{IE}}{dt}=\rho_{E}\rho_{I}\hat{K^{E}}+\rho_{E}w_{IE}\left(V_{Rexc}-\langle V^{I}\rangle \right)\bar{K_{+}^{E}\gamma^{E}}\\ \frac{dw_{II}}{dt}=\rho_{I}^{2}\hat{K^{I}}-\rho_{I}w_{II}\left(\langle V^{I}\rangle -V_{Rinh}\right)\bar{K_{+}^{I}\gamma^{I}} \end{array}$$

From the above equations, it is straightforward to see when $\hat{K^{E}}\;:\;={\bar{K}}_{+}^{E}-{\bar{K}}_{-}^{E}<0$ and $\overset{-}{K^{I}}:={\bar{K}}_{+}^{I}-{\bar{K}}_{-}^{I}>0$, the stationary solutions satisfy:


(14) $$\begin{array}{l} \frac{c_{EI}^{st}}{c_{EE}^{st}}=\frac{\hat{K^{I}}\bar{K_{+}^{E}\gamma^{E}}}{\hat{K^{E}}\bar{K_{+}^{I}\gamma^{I}}}=\frac{c_{II}^{st}}{c_{IE}^{st}} \end{array}$$

We take the proportion of inhibitory synapses to excitatory synapses to be equal for both excitatory and inhibitory neurons, i.e., $\frac{k_{EI}}{k_{EE}}=\frac{k_{II}}{k_{IE}}$. The above condition brings the slopes of the excitatory and the inhibitory nullclines close to each other (Equation 6) leading to the intersection in the semi-linear regime and proportionality of excitation and inhibition:


(15) $$\begin{array}{l} \frac{\rho_{I}^{st}}{\rho_{E}^{st}}\approx \frac{c_{EE}}{c_{EI}} \end{array}$$

**Figure 3:**
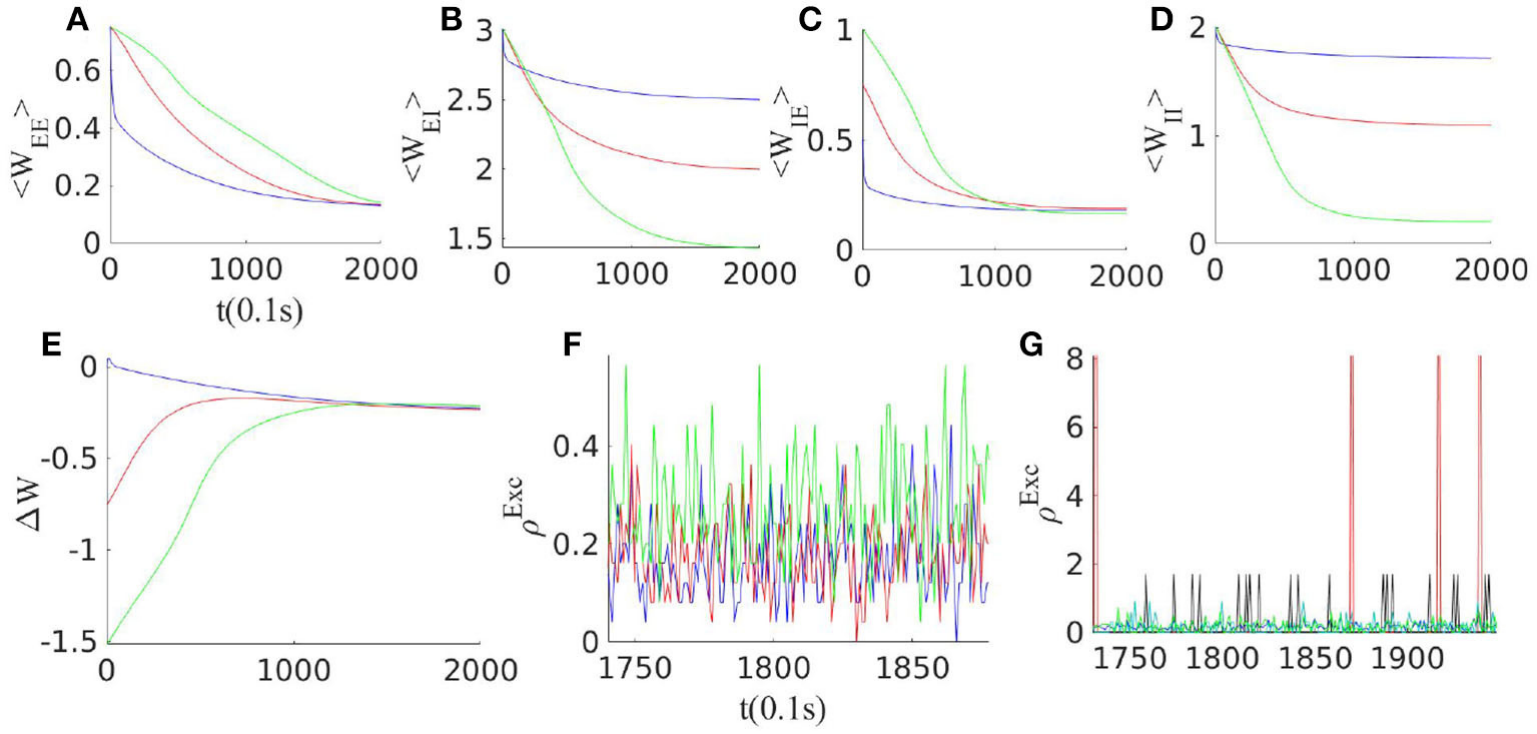
Effect of synaptic plasticity on the network with three different initial weight configurations when external excitatory input to both excitatory and inhibitory populations are of the same magnitude ρ_*Ext*_ = 150 Hz. **(A–D)** Evolution of average synaptic weights by STDP. **(E)** Change in the balance condition by STDP. Slopes of the Exc. and the Inh. nullclines approach each other under STDP in all three configurations. **(F)** The final state of the average neuron firing rates for these three networks lie below 1Hz. **(G)** Network activity for different clusters of neurons with different overall average inward synaptic weights. As STDP leads to an increase in the variance of the weight distribution, clusters with different overall connectivity strengths and correspondingly different average rates emerge.

Figure 3 shows that STDP tunes the weights to this balanced inhibition dominated state in which Δ*W* = *C*_*EE*_*C*_*II*_ − *C*_*EI*_*C*_*IE*_ ≈ 0. As the system settles in this state, synaptic plasticity has a strong effect when neurons are in a higher (here the linear) firing regime. In this state, rates vary co-linearly according to the above equation. On the other hand, synaptic plasticity rules for *w*_*II*_ and *w*_*EI*_, i.e., the second and fourth lines in equation (14), lead to a relation for stationary weights in the form of $\frac{c_{II}}{c_{EI}}=\frac{k_{II}\rho_{I}^{st}}{k_{EI}\rho_{E}^{st}}$. Comparing these last two equations, we arrive at $k_{II}c_{EE}^{st}=k_{EI}c_{II}^{st}$. Assuming *k*_*II*_ = *k*_*EI*_, the mentioned relation adjusts the trace of the Jacobian at the fixed point in the linear section to be near zero. Therefore, the plasticity rule and the dynamics of the near-linear regime stabilize the system near the BT point in the long term. When the external input to Exc. and Inh. populations are finely tuned, STDP alone can lead to the emergence of avalanches in the system. In the following section, by introducing a short-term plasticity mechanism we aim to tune the system at the critical point in the presence of fluctuating input.

### 3.2. Short-term plasticity tunes the network in a wide range of external input

In the following, first, we will discuss the adaptive role of short-term synaptic plasticity in bringing the network of the EI population to the avalanche regime. Afterward, we will discuss how internal or external noise close to the BT point can also cause the switch between the quiescent (Down) and the low firing (Up) states. We will discuss the Up-Down state transition by short-term depression can be achieved either through a switch between bi-stable states or by bringing the system close to the BT point by dampening the overall excitation. Here, we just consider the short-term plasticity of synapses between excitatory neurons. This type of plasticity might occur in other types of synapses as well, but we will not discuss this here. Because there exist numerous input synapses and we have assumed homogeneous connectivity, each neuron senses a large sample of the network activity and is connected with an overall average weight with a small variance to the excitatory neuron pool. With regard to these assumptions and structural homogeneity and based on Equation (8), we can write down the dynamic of the average synaptic weights to the neuron *i* in the state of the network with an excitatory population firing rate of magnitude ρ_*E*_ as:


(16) $$\begin{array}{l} \frac{dw_{EE}}{dt}=\frac{w_{EE}^{0}-w_{EE}\left(t\right)}{\tau_{STP}}-w_{EE}\left(t\right)q\rho_{E}\left(t\right) \end{array}$$

The rate equations for the EI population are of the form:


(17) $$\begin{array}{l} \frac{d\rho_{E}}{dt}=-\frac{1}{\tau_{m}}\left(\rho_{E}\left(t\right)-f\left(\rho_{E}\left(t\right),\rho_{I}\left(t\right),w_{EE}\left(t\right)\right)\right)\\ \frac{d\rho_{I}}{dt}=-\frac{1}{\tau_{m}}\left(\rho_{I}\left(t\right)-g\left(\rho_{E}\left(t\right),\rho_{I}\left(t\right)\right)\right) \end{array}$$

Taking the time scale of short term plasticity to be much larger than the EI-network activity decay time constant, i.e., τ_*STP*_ >> τ_*m*_, we can rewrite the dynamic in terms of fast, *d*/*dt*^*f*^, and slow time, *d*/*dt*^*s*^, evolution. Here, *t*_*f*_ = *t*/τ_*m*_ and *t*_*s*_ = *t*/τ_*STP*_. Defining μ = *t*_*s*_/*t*_*f*_ and ρ = $\left(\begin{array}{c} \rho^{E}\\ \rho^{I} \end{array}\right)$, we arrive at


(18) $$\begin{array}{l} \;\frac{d\rho }{dt^{f}}=-\left(\rho -f\left(\rho ,w_{EE}\right)\right)\\ \frac{dw_{EE}}{dt^{f}}=\mu \left(w_{EE}^{0}-w_{EE}\right)-q\tau_{m}w_{EE}\rho^{E} \end{array}$$

This set of equations can have a stable fixed point or an oscillatory behavior.

The average synaptic efficacy in the stationary state with the average excitatory rate $\rho_{E}^{*}$ is:


(19) $$\begin{array}{l} {\langle w_{EE}\rangle }_{St}=\frac{w_{EE}^{0}}{1+\tau q\rho_{E}^{*}} \end{array}$$

**Figure 4:**
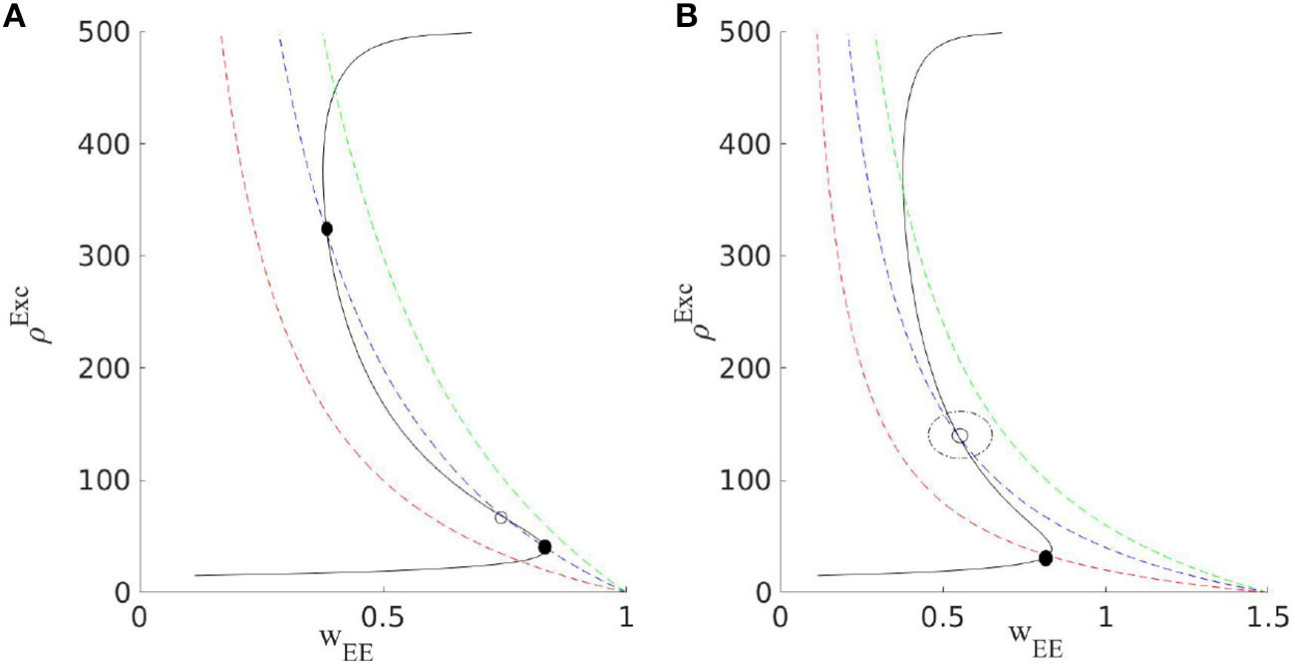
Output excitatory rates as a function of *W*_*EE*_ and the corresponding graphs for the average synaptic efficacy 〈*W*_*EE*_〉_*St*_ at three values of *q* (dashed red curve belongs to the largest and the dashed green curve is for the lowest value). Based on the value of $W_{EE}^{0}$, two different scenarios can occur. In **(A)**, by decreasing *q*, through a saddle-node bifurcation stable and unstable fixed points appear at low and high values of the rates. In **(B)**, with higher $W_{EE}^{0}$, by decreasing *q* after the Hopf bifurcation of the low firing rate fixed point, an oscillatory solution for (*u*, ρ_*out*_) emerges.

In case there exists a fixed point or a stable limit cycle solution around this point in the (ρ_*E*_, ρ_*I*_, 〈*W*_*EE*_〉_*st*_) phase space, the system might settle down at this solution (Figure 4A). The dynamics of the EI population near this region (with the slow-fast assumption) can be written as


(20) $$\begin{array}{l} \rho_{E}^{st}=f\left(k_{EE}\rho_{E}^{st},k_{EI}\rho_{I}^{st},\lambda_{E}^{Ex},\lambda_{I}^{Ex},\frac{w_{EE}^{0}}{1+\tau q\langle \rho^{st}\rangle }\right)\\ \rho_{I}^{st}=g\left(k_{EI}\rho_{E}^{st},k_{II}\rho_{I}^{st},\lambda_{E}^{Ex},\lambda_{I}^{Ex}\right) \end{array}$$

**Figure 5:**
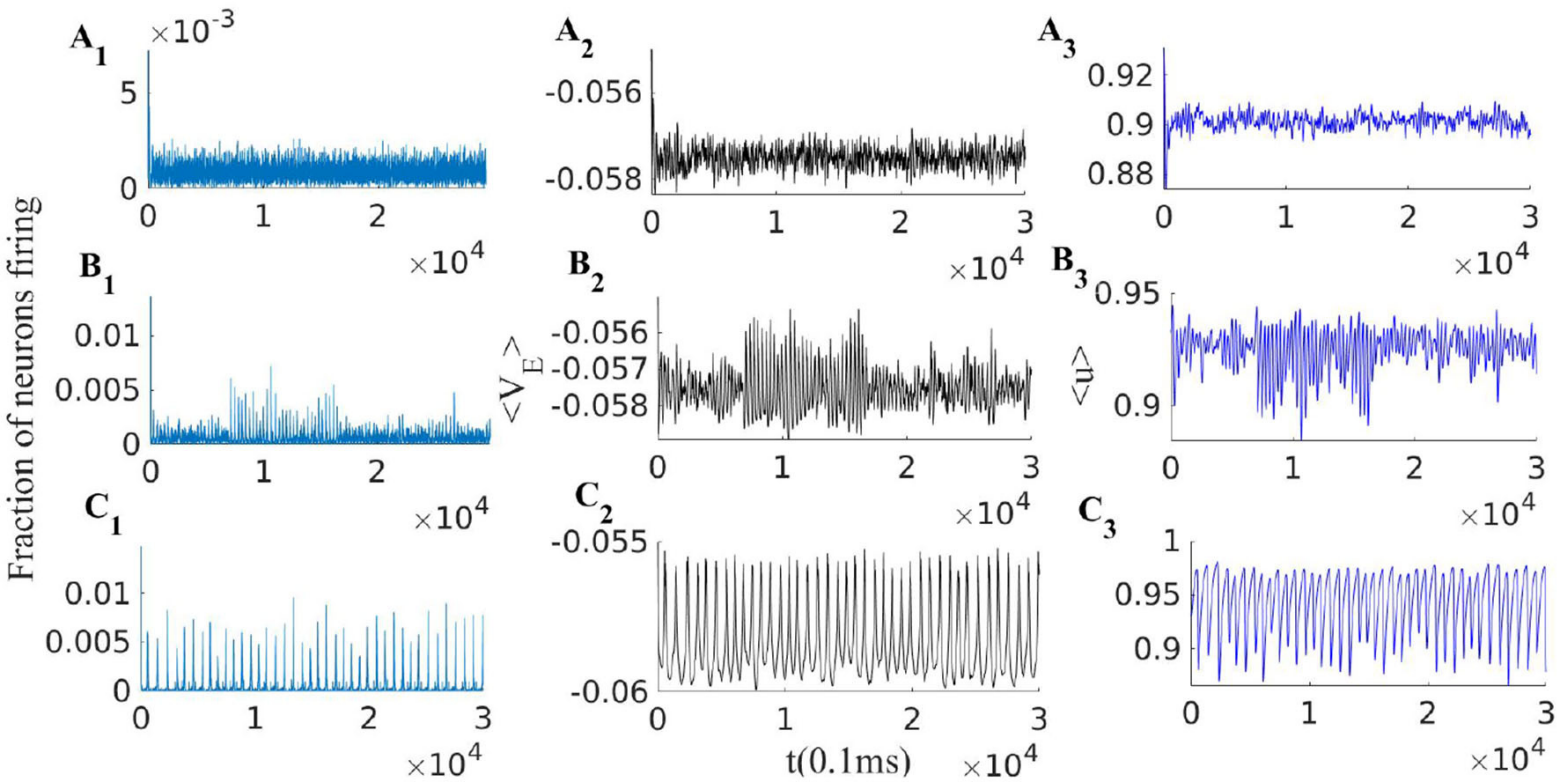
EI population with short term plasticity subjected to three different external excitatory rates $\rho_{Ext}^{exc}$ = [380, 280, 240]Hz in panels **(A1–A3)**, **(B1–B3)**, and **(C1–C3)** respectively. Other parameters of the model are *W*_*EI*_ = 1.5, *W*_*II*_ = 2, *W*_*IE*_ = 0.75, $W_{EE}^{0}=0.54$, $\rho_{Ext}^{inh}$= 150Hz, *q* = 0.3 and τ_*STP*_ = 10 * τ_*syn*_. Left plots **(A1, B1, and C1)**, show the excitatory population rates, middle plots **(A2, B2 and C2)**, show the population average membrane potential and right plots **(A3, B3 and C3)**, show the average excitatory synaptic efficacy 〈*u*〉.

This mechanism is effective to bring the system close to the BT point. Short synaptic plasticity is a method of gain control that can bring the system from a wide range of input and initial states to the low activity background state. In Figure 5, we consider a system that is already tuned by STDP to the balanced state of weights (Equation 14) receiving various rates of external excitatory input to the excitatory population. In all cases, the system is initially away from the BT point. Without STP, the system shows a high firing rate oscillatory activity with an average rate of around 300*Hz* (with Nullcline configuration of **Figure 7C**). STP brings all of them closer to the avalanche regime. The average synaptic efficacy 〈*u*〉 in these cases does not oscillate significantly.

**Figure 6:**
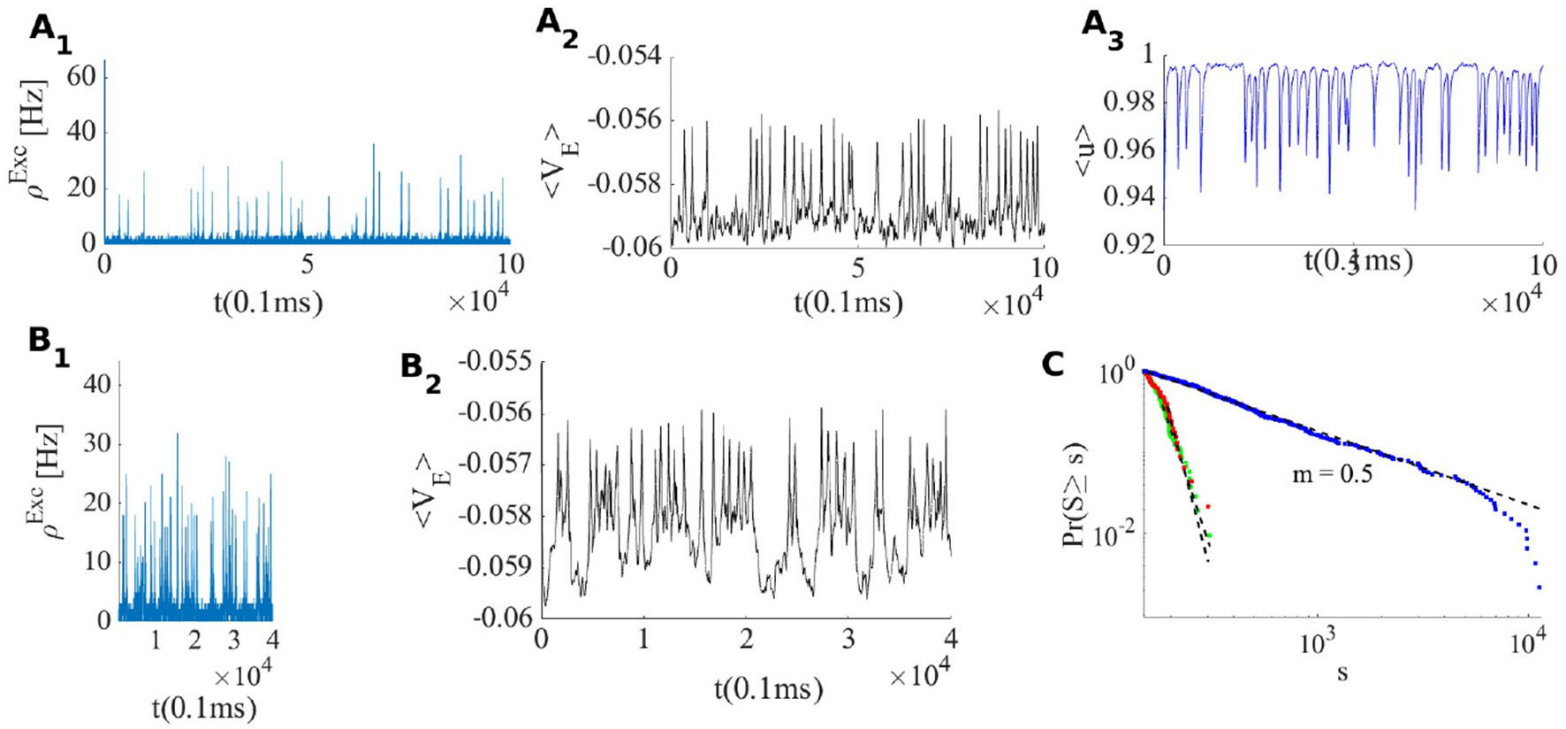
**(A)** System with the same parameters values as in Figure 5 except that here $\rho_{Ext}^{exc}=230Hz$. **(B)** EI population with the same parameters as in **(A)** but without STP. **(C)** Avalanche size distribution in a log-log plot for the network in **(A)** (blue) and two networks with smaller $W_{EE}^{0}$ which does not show scale free avalanches.

**Figure 7:**
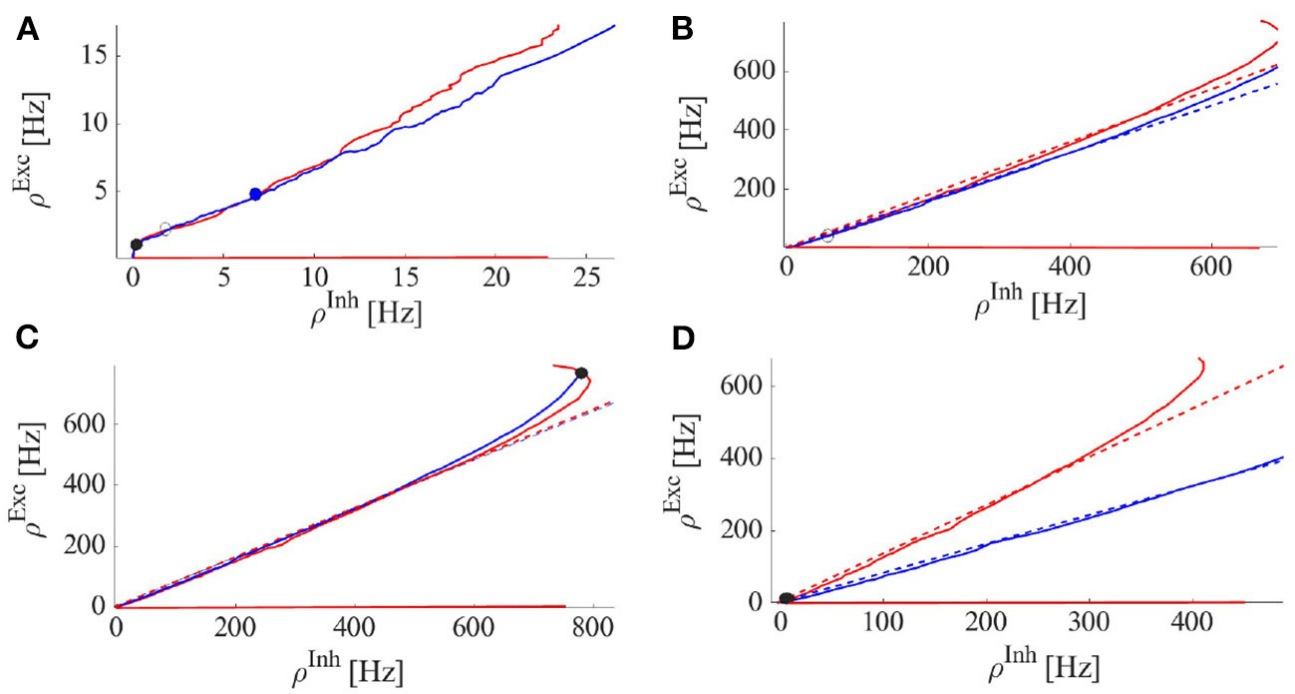
Excitatory (red) and inhibitory (blue) nullclines, i.e., solutions to Equation 4 for excitatory and inhibitory rates, respectively. Dashed lines are the linearized nullcline approximation with slopes of $k\frac{c_{EI}}{c_{EE}}$ (Exc.) and $k\frac{c_{II}}{c_{IE}}$ (Inh.) where k is a constant. STDP brings these slopes close to eachother (Equation 14). **(A)** Nullclines at the Avalanche regime: The volume of the basin of attraction of the stable quiescent state is small and a weakly stable or unstable fixed point at the intersection in the semi-linear regime of nullclines is close to the origin. **(B)** System with only a saddle fixed point with a limit cycle solution with medium firing rates. **(C)** System with only a high firing fixed point. **(D)** System with only a stable quiescent state fixed point.

When the external input rate is tuned very close to the *BT* point, where the quiescent fixed point and a low firing weakly (un)stable point lie close to each other, we see asynchronous avalanches of highly variable sizes (refer to Figures 6A, 7A). Without STP, we observe higher rates and less variable quiescent (Down) state time intervals (Figure 6B). Finite-size fluctuations kick the system out of the quiescent fixed point while STP plus fluctuations at the low rate fixed point drive the system back to the quiescent state. Average membrane potential and single neuron potentials in this case show a transition between two levels (refer to Figure 6B).

**Figure 8:**
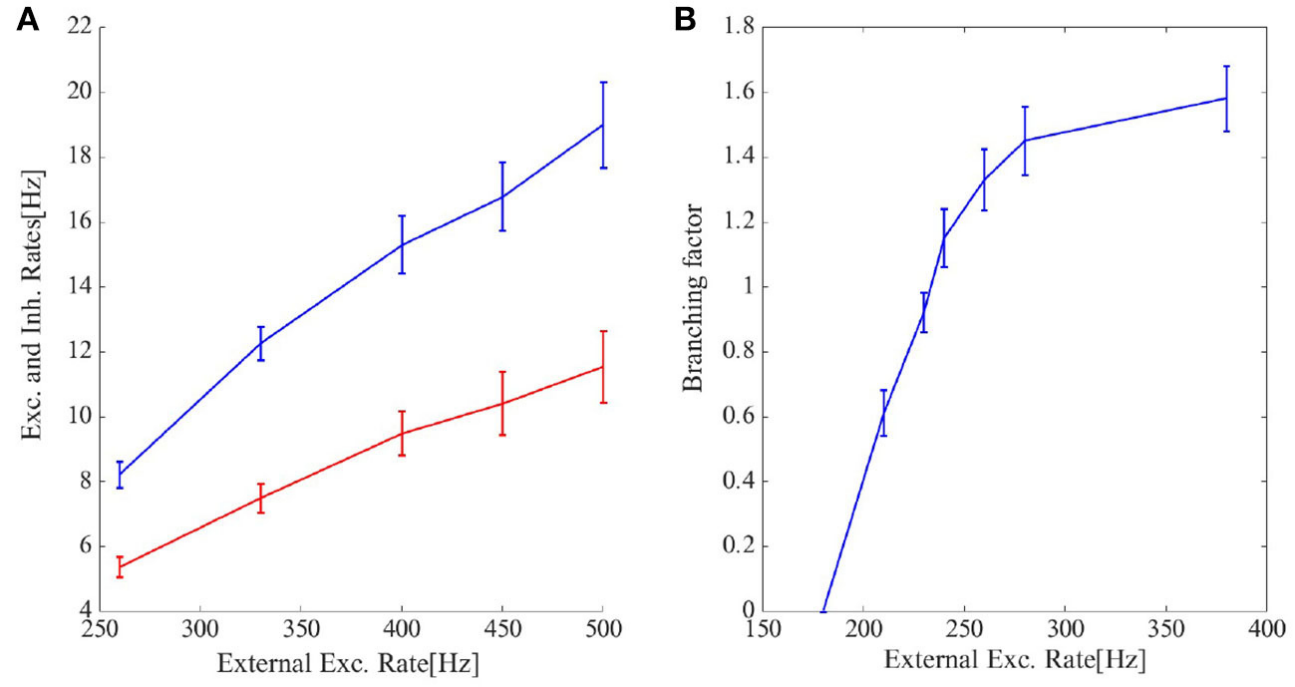
**(A)** The final excitatory (red) and inhibitory (blue) output rates for the system in Figures 5, 6A. STP works as a gain control mechanism. **(B)** The branching ratio in the network states shown in Figures 5, 6A is defined as the average number of post-synaptic neurons of a single presynaptic neuron which set to fire by receiving the presynaptic input spike. Higher external rates set more neurons close to the threshold and thus the branching factor increases. When the inflection point of the steady membrane potential distribution passes the membrane threshold in the steady-state firing rate regime this increment rate slows down leading to the concavity of the branching factor curve.

Figure 6C shows the cumulative avalanche size distribution function in a log-log plot for avalanches as in Figure 6A. The slope of the linear regression line is close to 0.5 indicating that the avalanche size distribution function is a power law with the exponent τ = −1.5. In the Appendix 1, we have presented statistics of the avalanches including duration distribution function, shape collapse, and power law scaling of mean size vs. mean duration. The branching ratio for the final state of the system is shown in Figure 8B. For ρ_*Ext*_ = 230*Hz*, the branching ratio is slightly less than one which is in agreement with our prediction in the avalanche regime. Figure 8A shows excitatory and inhibitory stationary rates of the EI population subject to external rates in the range 200–500 Hz. STP leads to low firing rate states and prevents overactivation.

**Figure 9:**
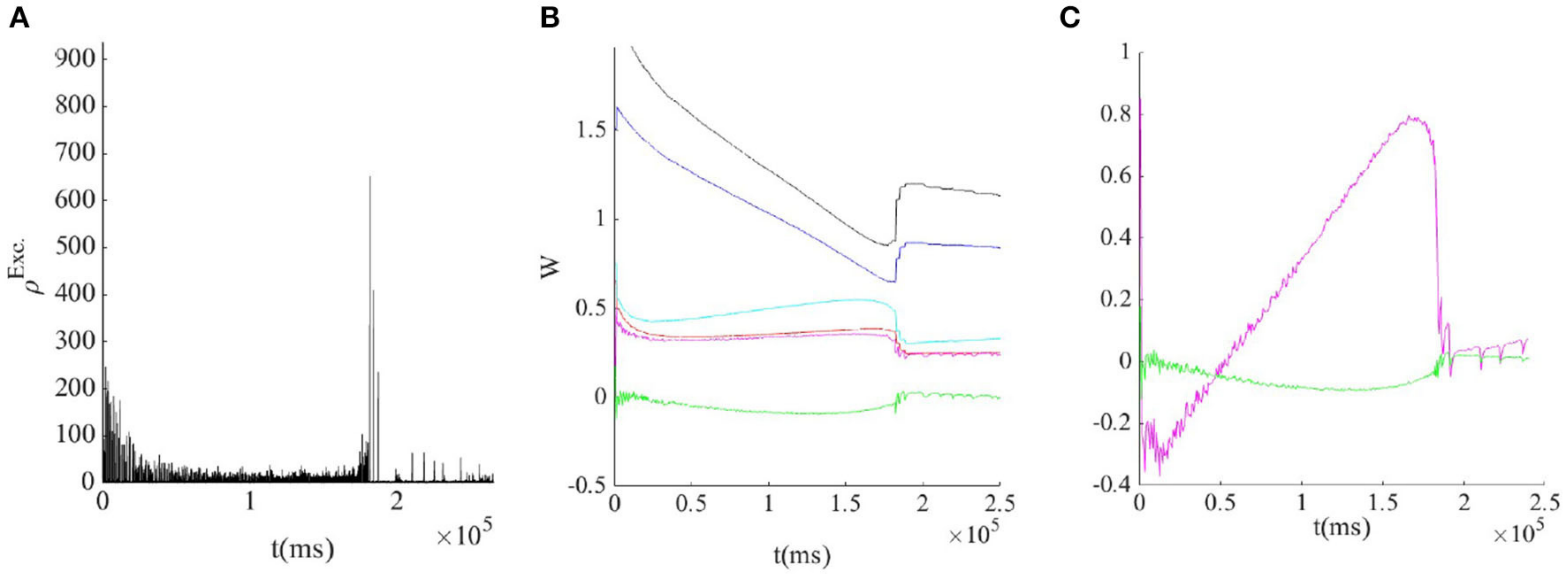
**(A)** STDP and STP tune the weights to the avalanche regime by tuning the inhibitory feedback. **(B)** Evolution of average weights: *W*_*II*_ (black),*W*_*EI*_ (blue),*W*_*IE*_ (cyan), *W*_*EE*_ (red), $W_{EE}^{eff}=uW_{EE}$ (magnet), and $\Delta W=W_{EE}^{eff}W_{II}-W_{EI}WIE$ (green). **(C)** In the avalanche regime, both the determinant(green) and the trace(magnet) of the Jacobian of the fixed point at the linearized nullclines are nearly zero.

As the system resides closer to the low firing regime, the effective synaptic strength of Exc.-Exc. connections is lowered by STP (Figure 7B). On the longer time scale STDP transforms the combination of weights to move the system to the avalanche regime by aligning the slopes of linearized nullclines and setting the trace of Jacobian at the fixed point on a linear section close to zero. Figure 9, shows the cooperation of both STDP and STP brings the system initially away from the BT point to the critical avalanche regime. STP prevents high firing as well as being trapped in the quiescent state, therefore constraining the system to be in a low firing state which in a longer time scale leads to tunning in the vicinity of BT point by STDP.

**Figure 10:**
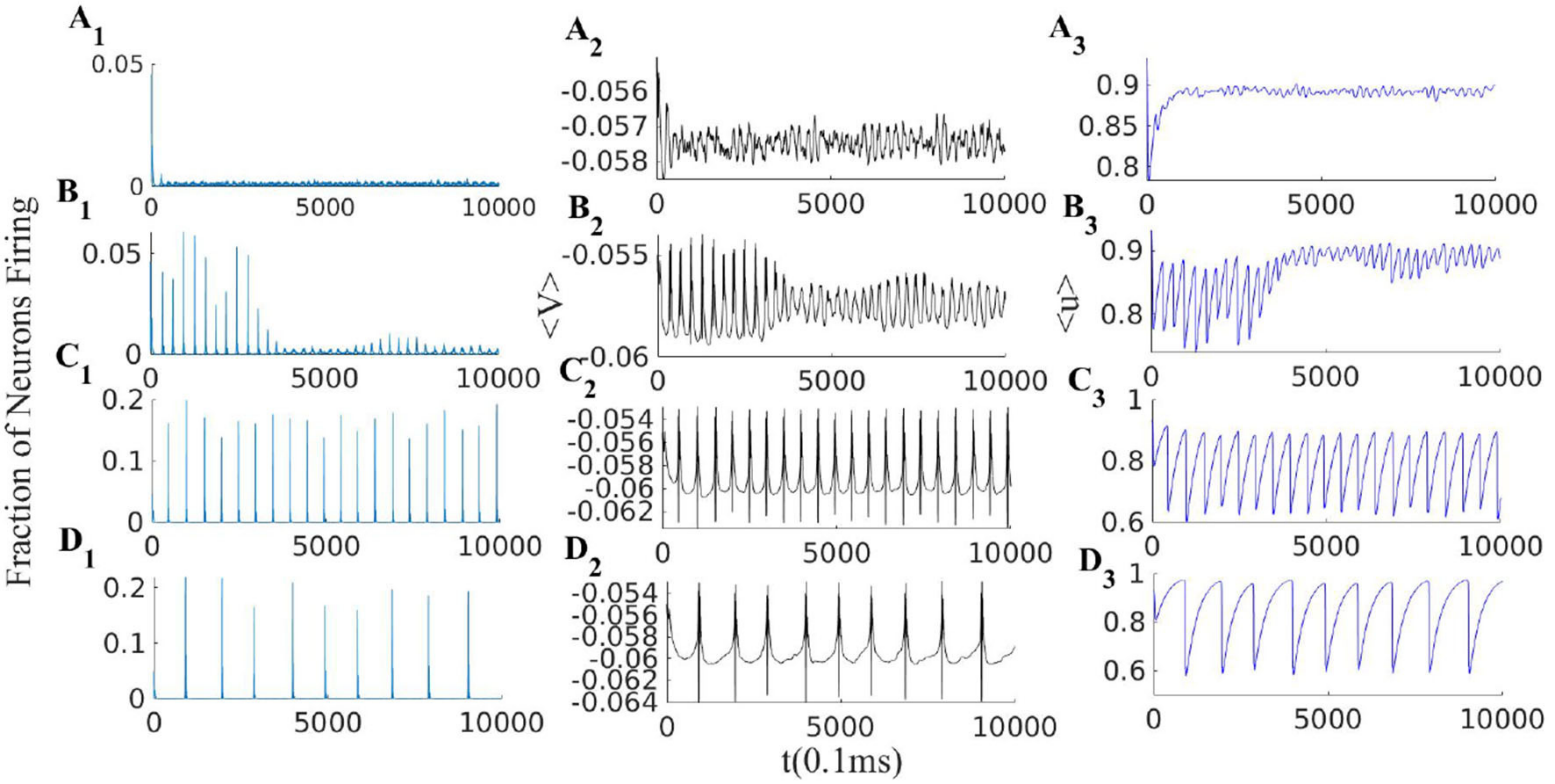
EI population with short term plasticity subjected to four different external excitatory rates $\rho_{Ext}^{exc}$ = [400, 330, 270, 225]Hz in panels **(A1–A3)**, **(B1–B3)**, **(C1–C3)**, and **(D1–D3)** respectively. Other network parmeters are *W*_*EI*_ = 2, *W*_*II*_ = 2, *W*_*IE*_ = 0.75, $W_{EE}^{0}=0.74$, $\rho_{Ext}^{inh}$ = 150 Hz and STP parameters are *q* = 0.4 and τ_*STP*_ = 10 * τ_*syn*_. Left plots **(A1–D1)**: excitatory rates; middle plots **(A2–D2)**: average membrane potential; right plots **(A3–D3)**: average synaptic efficacy 〈*u*〉.

Another way that STP can cause a switch between two distinct firing states is in the EI population which possesses bi-stability. In this case, a change of *u* can make each of the bi-stable nodes unstable while the system resides near them. The decrease of *u* in the up-state makes the up-state fixed point unstable at some value of *u*(*t*) (and accordingly *w*_*EE*_). Therefore, the system will jump to the remaining stable fixed point in a low or quiescent state. In the very low firing regime (the quiescent state), *u* will recover to its asymptotic value, and the average synaptic weight increases toward $w_{EE}^{0}$. If the quiescent state is unstable when *u* approaches its maximum value, we observe a transition to the high state. Moreover, if the volume of the basin of attraction of the quiescent fixed point is small, external and internal noise can also induce the transition to the high rate fixed point and the quiescent fixed point need not become unstable at $w_{EE}^{0}$. For high values of *u*, the up-state fixed point is the stable point of the fast system but an unstable point of the slow one. Therefore, following the slow path up-state loses stability and the fast system remains with only a stable low fixed point. The trajectory of the slow *u* is oscillatory in this case. Figure 10 shows both ways that STP can produce synchronous avalanche behavior in the system. When $W_{EE}=w^{0}$, the system is close to the constraints on the alignment of the semi-linear segments of the EI-nullclines which results in the presence of a high firing state as a unique fixed point of the system. In the high input rate case, ρ_*Ext*_ = 400*Hz*, corresponding to Figure 10A and the nullcline diagram of Figure 7C, due to STP, the system moves from a high state of activity to a limit cycle solution at lower firing rates. This final state is shown in Figure 10A and nullcline arrangements in this state are depicted in Figure 7B. Here, there is an unstable source in the linear branch sector which is surrounded by a limit cycle. Moreover, oscillations in 〈*u*〉 have a low amplitude because of the temporal averaging. On the other hand, the two bottom panels in Figure 10 are related to the situation of a switch between the high fixed point and the quiescent node. As shown in nullcline graphs in Figure 7C, at high synaptic efficacy the high firing state is the only stable fixed point, however, a high firing rate leads to a fast decline of the synaptic efficacy which brings the system to the state with the nullcline map of Figure 7D which has a stable quiescent fixed point. The final state activity, in this case, is composed of avalanches with a high rate of firing in a short time window. Decreasing the *q* factor can result in a longer up-state period. Also, 〈*u*〉 oscillates between two limits in these cases.

In this particular case, necessary conditions for up to down transitions are:


(21) $$\begin{array}{l} k_{EE}W_{EE}^{0}>k_{EI}W_{EI}\\ k_{EE}\frac{w_{EE}^{0}}{1+\tau q\rho^{H}}<k_{EI}W_{EI} \end{array}$$

The first condition means that the slope of the excitatory nullcline is smaller than the inhibitory one, which indicates a stable high firing fixed point. The second condition states that at this high firing state the stationary weight is not accessible before a stability loss. The slope of nullclines increases by the decrease of effective *W*_*EE*_ which causes the high state to lose stability either through a Hopf or a saddle-node bifurcation.

Finally, the plots in Figure 10B depict the case where STP brings the system close to the BT point which shows low to medium size avalanches with higher variability. In summary, the transition from a quiescent state to a high firing state can be of two distinct types. One way is that by increasing *W*_*EE*_, the quiescent fixed point and the unstable saddle move toward each other and in this way the basin of attraction of the quiescent fixed point shrinks and noise can initiate the escape from this fixed point to the higher firing state. This is the mechanism for alternation between quiescent periods and avalanche periods, corresponding to nullclines arrangements of Figures 7A,D with dynamics of Figure 6A. The other possibility is that a fixed point losses stability through a Hopf bifurcation either before or after the emergence of a saddle-node in the middle branch.

### 3.3. Continuum stochastic model of neuronal dynamics near BT point

We have shown that a single EI population in both regimes of sparse connectivity and all-to-all connectivity (Ehsani and Jost, 2022) exhibits scale-free avalanches near the critical point with Poisson firing of individual neurons. Next, we want to investigate if avalanche dynamics in weakly interconnected EI population persists. Our goal is to introduce a phenomenological stochastic field equation for the dynamics of population rates in the vicinity of the BT point.

We start with the model of Wilson and Cowan for the dynamics of the spatio-temporal mean fields of the excitatory and the inhibitory population rates, *E*(*x, t*) and *I*(*x, t*), in a 2D model of the cortex. Defining *V*(*x, t*)= $\left(\begin{array}{c} E\left(x,t\right)\\ I\left(x.t\right) \end{array}\right)$, *D* as the diffusion matrix and *f* : *R*^2^ → *R*^2^ as the gain function, one can write down the reaction-diffusion rates dynamics in the following form:


(22) $$\begin{array}{l} \tau \frac{\partial V\left(x,t\right)}{\partial t}=D\nabla^{2}V\left(x,t\right)+f\left(V\left(x,t\right)\right) \end{array}$$

The ODE part of this equation is the dynamic of a single EI population. The corresponding low firing fixed point (*E*_0_, *I*_0_) is stable in a specific parameter regime. It usually loses stability either *via* a saddle-node or a Hopf bifurcation which leads to either a region of bi-stability of low and high firing states or the emergence of oscillations. However, still far away from the bifurcation point since the diffusion matrix is not a scalar multiple of the identity, Turing instabilities can occur in the system.

Besides Turing instabilities, it can also happen that the fixed point itself loses its stability at *k* = 0 through a Hopf bifurcation, where the real part of the eigenvalues of *L* becomes zero:


(23) $$\begin{array}{l} \partial_{E}f_{E}+\partial_{I}f_{I}=0\\ det\left(L\right)=\partial_{E}f_{E}\partial_{I}f_{I}-\partial_{I}f_{E}\partial_{E}f_{I}>0 \end{array}$$

**Figure 11:**
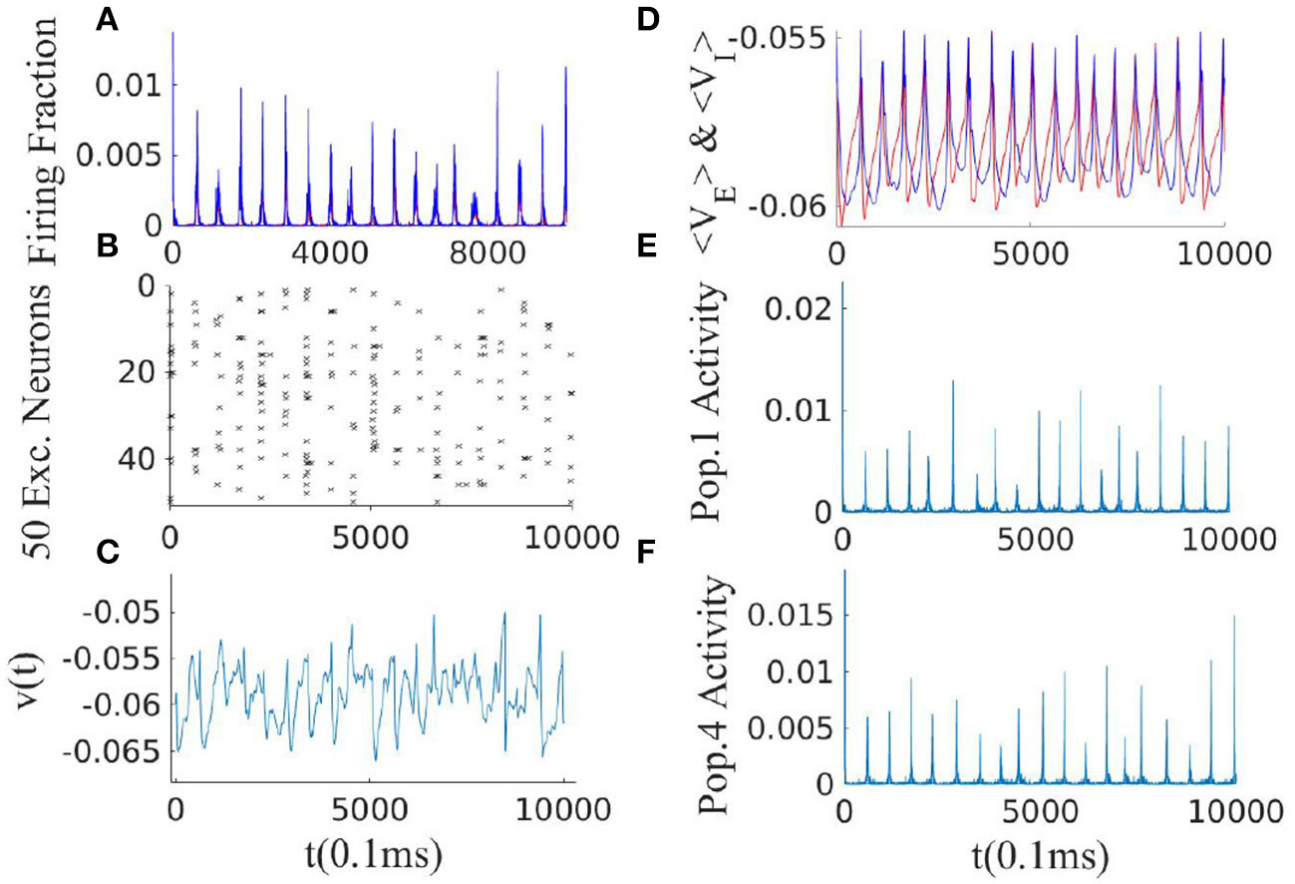
Simulation result of 20 interconnected EI populations each of size *N*_*E*_ = 10, 000 arranged on a ring. Average synaptic weights between two different EI subnetworks decay with the phase difference of them. Parameters of synaptic weights in each EI population are *W*_*EE*_ = 0.6, *W*_*EI*_ = 2, *W*_*II*_ = 2, *W*_*IE*_ = 0.75, $\rho_{Ext}^{E}=230Hz$, and $\rho_{Ext}^{I}=150Hz$. **(A)** The average firing rate of the whole network. **(B)** Raster plot of the sub-population of 50 neurons in one single EI subnetwork. Although neurons fire at avalanche times, they do not fire in all of them, which leads to high variability in their interspike interval times. **(C)** The membrane potential of a single neuron is in a constant transition between a state close to the threshold and a state close to the resting potential. **(D)** The average membrane potential of an EI-population (red for Exc. and blue for Inh.). **(E,F)** The activity of two distinct EI subpopulations shows high variability in the sizes of avalanches in both of them.

Furthermore, exactly at the BT point, we would also have *det*(*L*) = 0. Figure 11 shows the activity of 20 interconnected EI-populations each operating close to the BT point. Overall activity in this system is of synchronous avalanches type. Up-down state transitions also become synchronized. We can model weakly interconnected EI populations in the avalanche regime which shows oscillation of frequency ω_*i*_ as pulse-coupled oscillators and therefore investigate conditions on synchronization and traveling wave solutions. This analysis is out of the scope of the current work. Another approach consists in supplementing the macroscopic field equation with an appropriate noise term to derive the mesoscopic equation. As can be seen from Figure 11, the overall network activity is of avalanche type. This provides again evidence that avalanches are scale-free and occur in different temporal and spatial scales. However, our neural field model still lacks the internal finite-size fluctuation effects, inhomogeneities, and cross-correlation between individual neurons, and also inter-populations correlations.

To investigate the fluctuations around mean field rate dynamics, we study a Markov model inspired by the fact that close to the BT point, the firing of individual neurons is a Poisson point process. We consider a homogeneous network of size *N* in which temporal and spatial variances in the firing rates of neurons are minimal. In this network, fluctuations in the finite system firing rates in the steady-state will be proportional to $O\left(\frac{1}{\sqrt{N}}\right)$. To model the finite-size stochastic effects, we need to write down dynamics of micro-state evolution that match the mean-field upon coarse-graining. As we have seen, the operating region of the EI population is around a low firing state where neurons fire with high variability of inter-spike intervals indicating that we can model their spiking as a Poisson process. In this regime of activity, we can write down the microscopic evolution of a model neuron with two active and inactive states. The transition rate α between the active and the inactive state should model the vanishing of the postsynaptic potentiation, and the rate of inactive to active transition depends on the input and is, therefore, denoted by *f*(*i*). We want to model the system in the statistical homogenous state, in which the probability that a neuron fires depends only on the number of active neurons and therefore is the same for every neuron in the population.

After using system size expansion method (refer to Appendix 2), we arrive at the following equations for variance of population rate at the stationary point of the macroscopic equation:


(24) $$\begin{array}{l} \left(\begin{array}{ccc} A_{11} & 0 & A_{12}\\ 0 & A_{22} & A_{21}\\ A_{21} & A_{12} & A_{11}+A_{22} \end{array}\right)\left(\begin{array}{c} Var{\left(\epsilon \right)}_{st}\\ Var{\left(i\right)}_{st}\\ Cov{\left(\epsilon ,i\right)}_{st} \end{array}\right)=-\frac{\alpha }{2}\left(\begin{array}{c} \rho_{E}^{st}\\ \rho_{I}^{st}\\ 0 \end{array}\right) \end{array}$$

which has the solution:


(25) $$\begin{array}{l} Var{\left(\epsilon \right)}_{st}\approx c\left(\left(A_{11}A_{22}-A_{21}A_{12}+A_{22}^{2}\right)\rho_{E}+A_{12}^{2}\rho_{I}\right)\\ Var{\left(i\right)}_{st}\approx c\left(\left(A_{11}A_{22}-A_{21}A_{12}+A_{11}^{2}\right)\rho_{I}+A_{21}^{2}\rho_{E}\right)\\ Cov{\left(\epsilon ,i\right)}_{st}\approx -c\left(A_{11}A_{12}\rho_{I}+A_{21}A_{22}\rho_{E}\right) \end{array}$$

with $c=\frac{-\alpha }{2\left(A_{11}+A_{22}\right)\left(A_{11}A_{22}-A_{21}A_{12}\right)}$. The average population rate and the fluctuation around the macroscopic state are:


(26) $$\begin{array}{l} \langle \frac{E}{N_{E}}\rangle =\rho_{E}\;Var\left(\frac{E}{N_{E}}\right)=\frac{Var\left(\epsilon \right)}{N_{E}}\\ \langle \frac{I}{N_{I}}\rangle =\rho_{I}\;Var\left(\frac{I}{N_{I}}\right)=\frac{Var\left(i\right)}{N_{I}} \end{array}$$

From equations (25) and (26), it can be seen that close to the bifurcation of the macroscopic equation, i.e., the BT point, where both trace and determinant of the Jacobian are close to zero, fluctuation magnitudes increase. The important point is that the population variance is linear in the rates.

Moreover, close to the BT region, the fixed point on the semi-linear regime has an eigenvalue close to zero. Dynamics along the slow manifold which provides the detailed temporal balance of inhibition and excitation enables us to write the instantaneous inhibition rate as the linear function of the excitatory rate. Therefore, we can write down the rate dynamics of the excitatory population in terms of a stochastic field equation when both inhibitory feedback and fluctuations are local. From equation (15), we know that near the BT point, there is a linear relation between rates, i.e., $I\approx \frac{c_{EE}}{c_{EI}}E$. Therefore, the average current to the excitatory population close to the BT point (Equation 6) can be written as γ = *c*_*EE*_*E* − *c*_*EI*_*I* ≈ 0. The second derivative in the expansion of the gain function for the excitatory population from equation (22) in the region of low activity is


(27) $$\begin{array}{l} \frac{1}{2}\frac{\partial^{2}f}{\partial E^{2}}E^{2}+\frac{\partial^{2}f}{\partial EI}EI+\frac{1}{2}\frac{\partial^{2}f}{\partial I^{2}}I^{2} \end{array}$$

where we can use the following approximations for the gain function derivatives:


(28) $$\begin{array}{l} \frac{\partial^{2}f}{\partial I^{2}}\propto W_{EI}^{2},\;\frac{\partial^{2}f}{\partial E^{2}}\propto W_{EE}^{2},\;\frac{\partial f}{\partial I\partial E}\propto -W_{EE}W_{EI} \end{array}$$

By using the linear relation of the inhibitory and excitatory rates near the BT as the result of the projection of the dynamic to the slow manifold, we can replace inhibitory local field strength by a term linear in the local excitatory field. Besides, fluctuations in the average population activity, Equation (26), linearly depend on the rate. Therefore, we can write down the stochastic field equation for the excitatory rate in the region of small γ:


(29) $$\begin{array}{l} \frac{\partial E\left(x,t\right)}{\partial t}=\gamma E\left(x,t\right)+D\Delta E\left(x,t\right)-uE^{2}\left(x,t\right)+\psi \left(x,t\right)\\ \langle \psi \left(x,t\right)\psi \left(x^{\prime },t^{\prime }\right)\rangle =2\sqrt{\frac{\sigma^{2}}{N}}E\left(x,t\right)\delta \left(x-x^{\prime }\right)\delta \left(t-t^{\prime }\right) \end{array}$$

Here, *u* < 0 is the coefficient related to synaptic weights that can be explicitly derived by assuming a certain form of the gain function and proportionality of the rates. This stochastic partial differential equation after appropriate rescaling $E\left(x,t\right)=\frac{\sigma }{u\tau }S\left(x,t\right)$ agrees with the Langevin description of directed percolation which is of the following form with new transformed coefficients:


(30) $$\begin{array}{l} \frac{\partial S\left(x,t\right)}{\partial t}=\left(\gamma^{\prime }+D^{\prime }\Delta \right)S\left(x,t\right)-u^{\prime }{\;S}^{2}\left(x,t\right)+\psi \left(x,t\right)\\ \;\langle \psi \left(x,t\right)\psi \left(x^{\prime },t^{\prime }\right)\rangle =2u^{\prime }\;S\left(x,t\right)\delta \left(x-x^{\prime }\right)\delta \left(t-t^{\prime }\right) \end{array}$$

At γ′ = 0, the above system shows an absorbing state phase transition. Thus, from any active state, the system relaxes by avalanches with a power-law size distribution to an inactive state.

**Figure 12:**
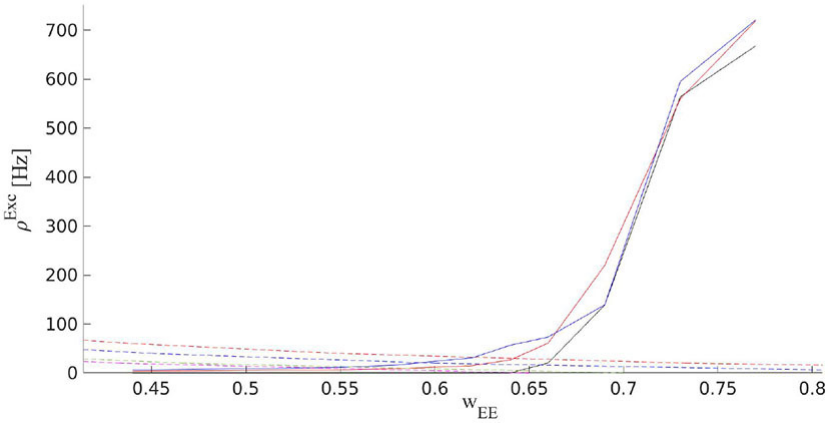
Solid curves are the output rates for three different external input strengths vs. *W*_*EE*_: Blue (400*Hz*), red (310*Hz*), and black (220*Hz*). Dashed curves show average stationary synaptic weight, 〈*W*_*EE*_〉, in the network with STP with different maximum synaptic efficacies: $W_{EE}^{0}=$1.3 (red), 0.9 (blue), 0.7 (green), and 0.65 (magenta). Intersections of the dashed and the solid curves are the fixed points of the EI network with STP for the corresponding control parameters. These fixed points are located in the low firing rate regime close to the avalanche region.

In an isolated EI population, external drive to the inhibitory and the excitatory population should be present to counterbalance the dissipation by the leaking currents and thereby set the average membrane potential in these neurons at a state above the resting threshold. External excitatory input to the excitatory population is slightly higher than the external drive to the inhibitory population which leads to a slightly higher average membrane potential in the excitatory population. Furthermore, we can assume that the external spike train to each neuron is Poisson as well. The external drive by itself would not lead to significant firing in the individual neurons but the strengths of the internal connections between them are tuned in a way that bursts of activity occur in the excitatory population which is then followed by the inhibitory ones. The internal feedback inhibition is strong enough to kill the excitatory burst. In a slightly inhibition-dominated regime, we have sharp synchronous responses to the external input in a short time window. On the other hand, the network has a safe margin from an overly active state. In the absence of the input distinguished from random external noise, the system shows scale-free avalanches because of the maintenance of the inhibition-excitation balance. However, the external drive must compensate for the dissipation of the system to stay at or near the critical point. Without mechanisms like short-term plasticity, the external drive has to be fine-tuned for the system to show criticality. However, short-term plasticity in a network in which synaptic weights are already near a slightly inhibition dominated regime broadens the range of the external drive strength which leads to critical avalanches. Figure 12 shows final output rates for three different values of the external excitatory rates and $W_{EE}^{0}$ with STP. In all these cases, the operating point of the system is close to the bifurcation point of the quiescent state.

We can extend the short term synaptic depression of equation (8) to a continuum field equation by defining a field of excitatory synaptic efficacy Ω(*x, t*) ∝ 〈*W*_*EE*_〉(*x, t*) with local dynamics of equation (16):


(31) $$\begin{array}{l} \tau_{m}\frac{\partial E\left(x,t\right)}{\partial t}=\left(-\alpha +\Omega \left(x,t\right)\right)E\left(x,t\right)+D\Delta E\left(x,t\right)\\ -uE^{2}\left(x,t\right)+\psi \left(x,t\right)\\ \frac{\partial \Omega \left(x,t\right)}{dt}=\frac{1}{\tau_{STP}}\left(\Omega_{0}-\Omega \right)-q\Omega E\left(x,t\right)\\ \langle \psi \left(x,t\right)\psi \left(x^{\prime },t^{\prime }\right)\rangle =2\sqrt{\frac{\sigma^{2}}{N}}E\left(x,t\right)\delta \left(x-x^{\prime }\right)\delta \left(t-t^{\prime }\right) \end{array}$$

**Figure 13:**
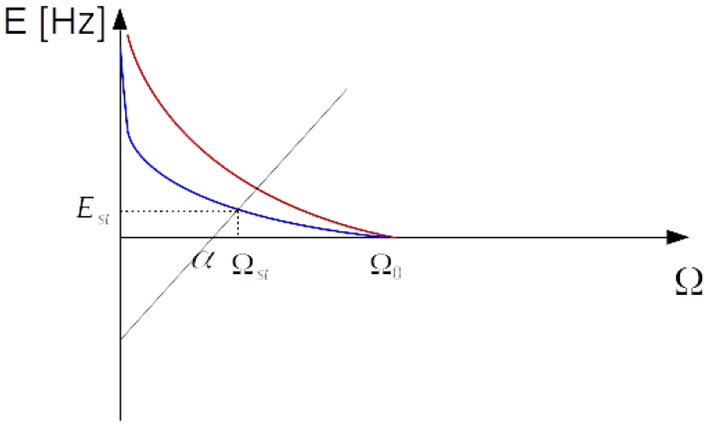
Intersection of curves given by equations (32) and (33). Setting Ω_0_ to a value close to α by STDP and sufficient amount of synaptic depression leads to a stationary value of Ω_*st*_ very close to the critical value. The blue curve is associated with a higher value of *q*.

Here, α represents both the decay of activity by leaky currents of the cells and the inhibition feedback which varies linearly with the excitatory rate. The dynamic excitatory synaptic strength brings the coefficient of the linear term to a value near zero (refer to Figure 13). This set of equations has a stationary synaptic efficacy solution of the value


(32) $$\begin{array}{l} \Omega_{st}=\frac{\Omega_{0}}{1+q\tau_{STP}E_{st}} \end{array}$$

On the other hand, in the active phase, the stationary homogeneous rate is :


(33) $$\begin{array}{l} E_{st}=\frac{-\alpha +\Omega_{st}}{u} \end{array}$$

Assuming |*u*| is a very small quantity and *E*_*st*_ is also small in the low firing rate regime then −α + Ω_*st*_ ≈ 0 and $E_{st}=\frac{1}{q\tau_{STP}}\left(\frac{\Omega_{0}}{\alpha }-1\right)$.

Long-term synaptic plasticity tunes Ω_0_ so that the coefficient of the linear term is close to zero and a moderate level of short-term depression suffices to bring the system to the critical point. Altogether, equation (31) is the description of an EI interconnected spiking neuron network tuned to the critical point of balancing inhibition and excitation both by long-term synaptic plasticity and short-term synaptic depression. The system wanders around the phase transition point and shows avalanche dynamics with scale-free size and time distribution, the proportionality of inhibition and excitation, up and down state transitions of the membrane potential and population activity rates, and oscillations of order of the 10*Hz* resembling ubiquitous alpha-band oscillations.

## 4. Discussion

In this study, we have proposed a self-organizing model for the cortical dynamics which tunes the system to the regime of low firing avalanche dynamics corresponding to the ongoing intrinsic activity in the cortex. We showed that long-term synaptic plasticity by STDP tunes the synaptic weights to achieve the internal balance of inhibition and excitation. This effect does not depend on the exact shape of STDP kernels, however, the stability of results depends on the sign of the integral of the kernels. On the other hand, short-term depression of excitatory synapses can tune the system in response to the wide range of the strength of the external drive. We have not considered short-term plasticity for other types of synapses. However, we believe that under appropriate conditions on their respective strengths, we can observe the same qualitative regulation effects. Short-term homeostatic regulations acting at the time scale of neuronal dynamics are needed to self-tune the open non-conservative system subjected to varied external input at the critical point. On the other hand, long-term plasticity is responsible for bringing the system close to the bifurcation point. The interplay of these two types of synaptic plasticity is responsible for self-tuning to the critical state.

At the critical state, our system shows power law distribution functions for avalanches size and duration which matches the branching process and mean-field directed percolation process. In addition, scaling relation among the exponents and avalanche shape collapse is another proof that we are at the edge of a second-order phase transition. We have shown in Ehsani and Jost (2022) that close to the BT point, the branching factor is close to one because of the tight balance of inhibition and excitation and Poisson firing of neurons in the avalanche regime.

Feedback inhibition is proportional to the excitatory rate on the slow manifold i.e., *I*(*t*) = α*E*(*t*) where $\alpha \propto \frac{W_{EE}}{W_{EI}}$. By using this linear relation between rates, we rewrite the Wilson-Cowan field equation in the regime of low firing rate where the system is locally close to the BT point. Moreover, we showed that internal noise in local EI populations in this state is of Poisson type. Therefore, by knowing the noise variance and the fact that there is a tight temporal balance of inhibition and excitation, we showed that the excitatory field dynamics formulated as a stochastic field equation in the low firing rate regime matches the Langevin description of directed percolation at the absorbing state phase transition. However, there is not such an absolute absorbing state in our system. External drive to the neurons, which is also stochastic, would lead to the background level of firing and fluctuations in the network. In an open system, the external load has to be fine-tuned to compensate for the dissipation to remain at the critical point. Short-term depression of excitatory synapses allows this tuning for a wider range of external drives. Tuning the system at the critical point can be achieved by coupling fast population dynamics with slow adaptive gain and synaptic weight dynamics, which make the system wander around the phase transition point. Therefore, by introducing short-term and long-term synaptic plasticity, we have proposed a self-organized critical stochastic neural field model (Equation 31).

We have shown that in a weakly interconnected neuronal mass model the avalanche dynamics persist when local subpopulations are close to the BT critical point. However, a comprehensive study of the coupled field equation dynamics in a 2D or 3D network requires further study. Types of solution and dynamic repertoire of this coupled stochastic field equations have not been done in this study. Neural field models, without noise, can show vast dynamical solutions, including wave propagation in terms of a front solution in a bistable network (refer to Amari, 1977; Ermentrout, 1998), propagating pulses in an excitable medium (refer to Pinto and Ermentrout, 2001b), and spatially localized oscillations, spiral waves in the oscillatory regime of a local EI population (Troy and Shusterman, 2007), localized bump solutions (Pinto and Ermentrout, 2001a) and spatially periodic patterns called Turing patterns (Ermentrout and Cowan, 1979 also refer to Bressloff, 2011, for a comprehensive review). Analysis of the full stochastic version of neuronal field formulated in terms of coupled or non-coupled SPDE is a rather new area of study. The stochastic version of the continuum neural field for the excitatory population without STP has been discussed in Buice and Cowan (2007) and Bressloff (2019). Buice and Cowan used a coherent path formalism and the Doi-Peliti functional representation to translate the microscopic master equation to a path integral representation for activity fields (Buice and Cowan, 2007). One advantage of their method is that the study of scale invariance at criticality in the functional representation is possible. They proposed that the stochastic neural field equation for the excitatory system at a critical point can be written in the form of Langevin's description of directed percolation. Using coherent path formalism to investigate the critical behavior of the coupled system of Equation (31) can be illuminative for understanding the critical behavior of the system. Moreover, we have written down the dynamics of the excitatory field assuming a fast linear inhibitory feedback. Modeling the whole dynamic as a directed percolation of two dynamical variables is another possibility that we have not explored.

## Data availability statement

The raw data supporting the conclusions of this article will be made available by the authors, without undue reservation.

## Author contributions

ME and JJ designed the research. ME performed research and wrote the manuscript. JJ edited the manuscript. All authors reviewed the manuscript, contributed to the article, and approved the submitted version.

## Funding

This study was funded by the International Max Planck Research School and the Max Planck Institute for Mathematics in Sciences in Leipzig.

## Conflict of interest

The authors declare that the research was conducted in the absence of any commercial or financial relationships that could be construed as a potential conflict of interest.

## Publisher's note

All claims expressed in this article are solely those of the authors and do not necessarily represent those of their affiliated organizations, or those of the publisher, the editors and the reviewers. Any product that may be evaluated in this article, or claim that may be made by its manufacturer, is not guaranteed or endorsed by the publisher.
