# Supplementary material for: Self-organized criticality in a mesoscopic model of excitatory-inhibitory neuronal populations by short-term and long-term synaptic plasticity
Source: Front Comput Neurosci. 2022 Oct 10;16:910735. doi: 10.3389/fncom.2022.910735 (PMC9588946; doi:10.3389/fncom.2022.910735)
Supplement: Supplementary file 1 [file Data_Sheet_1.pdf]

Appendices of : Self-organized criticality in a mesoscopic model of excitatory-inhibitory neuronal populations by short-term and long-term synaptic plasticity

Masud Ehsani[1*] and Jürgen Jost[1,2]

[1*] Max Planck Institute for Mathematics in Sciences, Inselstr.22, Leipzig, 04103, Saxony, Germany .
[2]Santa Fe Institute, 1399 Hyde Park Rd, Santa Fe, NM 87501, United States .

*Corresponding author(s). E-mail(s): masud.ehsani@mis.mpg.de; Contributing authors: jjost@mis.mpg.de;

1 Avalanche statistics

It is known that threshold level and duration of temporal bins affect avalanches' shape and size distributions. One can choose avalanches as periods of firing between two quiescent states or above a certain threshold level. In both cases, fluctuations during an avalanche can cause the return to the threshold and leads to detection of spurious avalanches. We define avalanches as distinct bursts of activity, therefore, it is necessary to exclude these sub-avalanches. To circumvent this problem, we perform a moving average with a window of two adjacent time bins on the population firing rate. Time window of moving average has the similar effect as temporal binning in the experimental results. Since we have synaptic delay of one temporal unit between neurons, it make sense to perform moving average of two time units. The width of temporal bins does not affect the slope of curves for large sizes of avalanches as it does not affect identifying the avalanches of large sizes and their relative frequencies. When temporal binning window is small enough we observe that big avalanches is

preceded and followed up by series of smaller avalanches. However, choosing large width for temporal averaging obscure this fact. Moreover, choosing small threshold value in a way to exclude single neurons firing while observing scaling in large avalanche sizes does not effect asymptotic exponents. We define avalanches as bursts of activity higher than a threshold which is around $0.2Hz$ corresponding to random background activity driven by external noise at the quiescent state. We select this value by averaging firing rates of neurons Using the algorithm introduced in Clauset and Newman (2009), we fit the power-law distribution to avalanches size and duration with the maximum likelihood estimator(MLE) algorithm adjusting for s_{min} which leads to the best power-law fit with the lowest Kolmogorov-Smirnov(KS) measure. We check the goodness of the fit and also compute directly the MLE from the following formula:

$$\alpha^* = 1 + N(\sum_i log(\frac{s_i}{s_{min}}))^{-1} \tag{1}$$

Near BT, we observe power-law scaling of size and duration of avalanches with exponents $\tau \sim 1.5$ and $\alpha \sim 2$. Average size versus average duration of avalanche is also a power-law with $< s >=< T >^{\frac{1}{\sigma\nu z}}$ followed by a scaling relation between exponents as $\frac{\alpha - 1}{\tau - 1} = \frac{1}{\sigma\nu z} = 2$. Moreover, the temporal profile of avalanches is described by a single universal scaling function:

$$S(t, T) \sim T^{1/\sigma\nu z - 1} F(t/T) \tag{2}$$

2 Finite size fluctuations in a single EI population

So far we have analyzed mean-field models which were based on neglecting finite system size, inhomogeneities in the synaptic connections, and stochastic effects. Far from bifurcations of the mean-field (MF) equations, the behavior of the average rates of the stochastic system follows predictions of MF accurately. In this case, basins of attraction of the fixed points are separated enough and stochastic effects do not lead to a change in the macroscopic behavior of the system. However, close to the bifurcation points of the macroscopic system, internal and external fluctuations can cause the stochastic system to evolve differently from predictions of MF models. For example, it can cause transitions between different fixed points. Let us consider a homogeneous network of size N in which temporal and spatial variances in the firing rates of neurons are minimal. In this network, fluctuations in the finite system firing rates in the steady-state will be proportional to $O(\frac{1}{\sqrt{N}})$. To model the finite-size stochastic effects, we need to write down dynamics of micro-state evolution that match the mean-field upon coarse-graining. As we have seen, the operating region of the EI population is around a low firing state where neurons fire with high

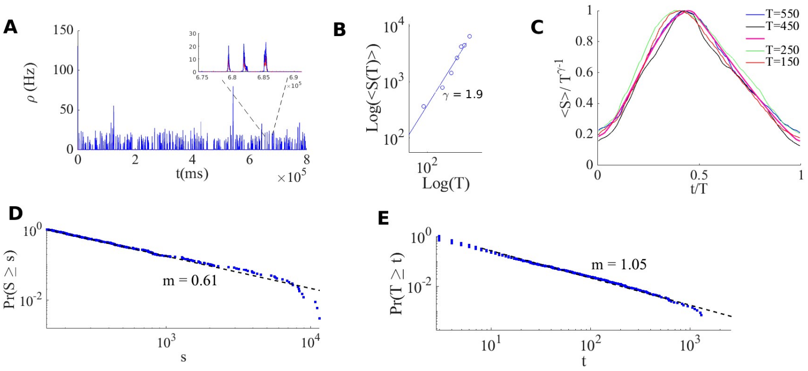

Fig. 1: (A) Excitatory(red) and Inhibitory(blue) population rates in the avalanches regime. Inhibitory instantaneous rate is proportional to the excitatory rate which leads to tight temporal balance. (B) Average size vs. average duration of avalanches. (C) Temporal profile of three sets of avalanches with different duration. (D) The cumulative distribution function of inhibitory avalanches size. (KS = 0.056) (E) Cumulative distribution function of inhibitory avalanche duration.(KS=0.068)

variability of inter-spike intervals indicating that we can model their spiking as a Poisson process. In this regime of activity, the Poisson neuron assumption enables us to write down the microscopic evolution of a model neuron with two active and inactive states. The transition rate α between the active and the inactive state should model vanishing of the postsynaptic potentiation, and the rate of inactive to active transition depends on the input and is therefore denoted by $f(i)$. We want to model the system in the statistical homogenous state, in which the probability that a neuron fires depends only on the number of active neurons and therefore is the same for every neuron in the population.

In the sequel, we consider a population of N_E excitatory and N_I inhibitory neurons, in which neurons change their states independently. Let us take $P(E, I, t)$ as the probability density of the EI population being in a state with E the number of active excitatory and I the number of active inhibitory neurons at time t. The following master equation describes the microscopic evolution of the system:

$$\begin{aligned}\frac{\partial P(E,I,t)}{\partial t} =& -\alpha[(EP(E,I,t)+IP(E,I,t)]\\&+\alpha[(E+1)P(E+1,I,t)+(I+1)P(E,I+1,t)]\\&+(N_E-E+1)f(c_{EE}(E-1),c_{EI}I)P(E-1,I,t)\\&-(N_E-E)f(c_{EE}E,c_{EI}I)P(E,I,t)\\&+(N_I-I+1)g(c_{IE}E,c_{II}(I-1))P(E-1,I,t)\end{aligned}$$

$$- (N_I - I)g(c_{IE}E, c_{II}I)P(E, I, t) \tag{3}$$

in which $c_{xy} = \dfrac{K_{xy}}{N_x} w_{xy}$, with K_{xy} being the number of incoming connections to a neuron in the population x from the population y , $f(.)$ and $g(.)$ are rates of inactive to active transtition for excitatory and inhibitory neurons,respectively. We use the system size expansion method Van kampen (2007) for truncating the moment hierarchy based on taking an ansatz on the order of the finite size fluctuation in the system. Assuming fluctuations around the deterministic (average field) trajectory to be of order $O(N)$, we can rewrite our stochastic variables in terms of a deterministic and a fluctuating term as

$$E = N_E \rho_E + N_E^{1/2} \epsilon, \qquad I = N_I \rho_I + N_I^{1/2} i \tag{4}$$

where ϵ and i are representing fluctuations around the deterministic trajectories. Defining $P(E, I, t) = Q(\epsilon, i, t)$, we can rewrite the l.h.s. of the master equation in terms of the new parameters as

$$\frac{\partial P(E, I, t)}{\partial t} = \frac{\partial(\epsilon, i, t)}{\partial t} - N_E^{1/2} \frac{d\rho_E(t)}{dt} \frac{\partial Q}{\partial \epsilon} - N_I^{1/2} \frac{d\rho_I(t)}{dt} \frac{\partial Q}{\partial i} \tag{5}$$

Defining ladder operators $Z_E f(E) = f(E + 1)$ and $Z_E^{-1} f(E) = f(E - 1)$ and expanding them in powers of ϵ , we arrive :

$$\begin{aligned} Z_E &= 1 + N_E^{-1/2} \frac{\partial}{\partial \epsilon} + \frac{1}{2} N_E^{-1} \frac{\partial^2}{\partial^2 \epsilon} + ... \\ Z_E^{-1} &= 1 - N_E^{-1/2} \frac{\partial}{\partial \epsilon} + \frac{1}{2} N_E^{-1} \frac{\partial^2}{\partial^2 \epsilon} + ... \end{aligned} \tag{6}$$

We define the same ladder operators for the inhibitory population states. Plugging all these equations into the master equation (3), we have:

$$\begin{aligned} &\frac{\partial Q(\epsilon, i, t)}{\partial t} - N_E^{1/2} \frac{d\rho_E(t)}{dt} \frac{\partial Q}{\partial \epsilon} - N_I^{1/2} \frac{d\rho_I(t)}{dt} \frac{\partial Q}{\partial i} = \alpha(Z_E - 1)[(N_E \rho_E + N_E^{1/2})\epsilon)Q] \\ &+ (Z_E^{-1} - 1)[N_E(1 - \rho_E - N_E^{-1/2}\epsilon) * f(c_{EE} N_E(\rho_E + N_E^{-1/2}\epsilon), c_{EI} N_I(\rho_I + N_I^{-1/2} i))Q] \\ &+ \alpha(Z_I - 1)[(N_I \rho_I + N_I^{1/2} i)Q(\epsilon, i, t)] \\ &+ (Z_I^{-1} - 1)[N_I(1 - \rho_I - N_I^{-1/2} i) * g(c_{IE} N_E(\rho_E + N_E^{-1/2}\epsilon), c_{II} N_I(\rho_I + N_I^{-1/2} i))Q] \end{aligned} \tag{7}$$

Expanding the inactive to active transition rates as

$$\begin{aligned} f(c_{EE} N_E(\rho_E + N_E^{-1/2}\epsilon), c_{EI} N_I(\rho_I + N_I^{-1/2} i)) =& f(c_{EE} N_E \rho_E, c_{EI} N_I \rho_I) \\ &+ N_E^{-1/2} \frac{\partial f}{\partial \rho_E} \epsilon + N_I^{-1/2} \frac{\partial f}{\partial \rho_I} i + ... \end{aligned} \tag{8}$$

Using the same expansion for ladder operators, we can sort the right and the left sides of equation (7) in powers of N_E and N_I . Equating terms of the order $O(N_E^{1/2})$ and $O(N_I^{1/2})$ leads to the macroscopic equation:

$$
\begin{aligned}
-\frac{d\rho_E(t)}{dt} &= \alpha\rho_E(t) - (1-\rho_E(t))f(\kappa_{EE}w_{EE}N_E\rho_E, \kappa_{EI}w_{EI}\rho_I) \\
-\frac{d\rho_I(t)}{dt} &= \alpha\rho_I(t) - (1-\rho_I(t))g(\kappa_{IE}w_{IE}N_I\rho_I, \kappa_{II}w_{II}\rho_I)
\end{aligned}
\tag{9}
$$

Equating terms of order $O(N^0)$ leads to a linear FPE for $Q(\epsilon, i, t)$ of the form:

$$
\begin{aligned}
\frac{\partial Q(\epsilon, i, t)}{\partial t} =& (\alpha - (1-\rho_E)\frac{\partial f}{\partial \rho_E} + f)\frac{\partial}{\partial \epsilon}\epsilon Q + (\alpha - (1-\rho_I)\frac{\partial g}{\partial \rho_I} + f)\frac{\partial}{\partial i} iQ \\
& - (1-\rho_E)\frac{\partial f}{\partial \rho_I}\frac{\partial}{\partial \epsilon} iQ - (1-\rho_I)\frac{\partial g}{\partial \rho_E}\frac{\partial}{\partial i}\epsilon Q \\
& + \frac{1}{2}(1-\rho_E)f\frac{\partial^2}{\partial \epsilon^2}Q + \frac{1}{2}(1-\rho_I)g\frac{\partial^2}{\partial i^2}Q
\end{aligned}
\tag{10}
$$

Defining matrices A and B as

$$
\begin{aligned}
\begin{pmatrix} A_{11} & A_{12} \\ A_{21} & A_{22} \end{pmatrix} &= \begin{pmatrix} -\alpha + (1-\rho_E)\frac{\partial f}{\partial \rho_E} - f & (1-\rho_E)\frac{\partial f}{\partial \rho_I} \\ (1-\rho_I)\frac{\partial g}{\partial \rho_E} & -\alpha + (1-\rho_I)\frac{\partial g}{\partial \rho_I} - g \end{pmatrix} \\
B &= \begin{pmatrix} (1-\rho_E)f & 0 \\ 0 & (1-\rho_I)g \end{pmatrix}
\end{aligned}
\tag{11}
$$

the amplitude of fluctuating term evolves as

$$
\frac{\partial}{\partial t}\begin{pmatrix} \langle\epsilon\rangle \\ \langle i\rangle \end{pmatrix} = \begin{pmatrix} A_{11} & A_{12} \\ A_{21} & A_{22} \end{pmatrix}\begin{pmatrix} \langle\epsilon\rangle \\ \langle i\rangle \end{pmatrix}
\tag{12}
$$

The covariance matrix $C = \begin{pmatrix} Var(\epsilon) & Cov(\epsilon, i) \\ Cov(\epsilon, i) & Var(i) \end{pmatrix}$ satisfies :

$$
\frac{\partial}{\partial t}C = AC + CA^t + B
\tag{13}
$$

If the determinant of A is positive and its trace is negative at the stationary point of the macroscopic equation (i.e., A has two negative eigenvalues), then the averages of the fluctuation terms go to zero. At the stationary point of the macroscopic equation, we have:

$$
\begin{pmatrix} A_{11} & 0 & A_{12} \\ 0 & A_{22} & A_{21} \\ A_{21} & A_{12} & A_{11} + A_{22} \end{pmatrix}\begin{pmatrix} Var(\epsilon)_{st} \\ Var(i)_{st} \\ Cov(\epsilon, i)_{st} \end{pmatrix} = -\frac{\alpha}{2}\begin{pmatrix} \rho_E^{st} \\ \rho_I^{st} \\ 0 \end{pmatrix}
\tag{14}
$$

which has the solution :

$$\begin{aligned}
Var(\epsilon)_{st} &\approx c((A_{11}A_{22} - A_{21}A_{12} + A_{22}^2)\rho_E + A_{12}^2\rho_I) \\
Var(i)_{st} &\approx c((A_{11}A_{22} - A_{21}A_{12} + A_{11}^2)\rho_I + A_{21}^2\rho_E) \\
Cov(\epsilon, i)_{st} &\approx -c(A_{11}A_{12}\rho_I + A_{21}A_{22}\rho_E)
\end{aligned} \tag{15}$$

with $c = \dfrac{-\alpha}{2(A_{11} + A_{22})(A_{11}A_{22} - A_{21}A_{12})}$.

The average population rate and the fluctuation arround the macroscopic state are:

$$\begin{aligned}
\langle \frac{E}{N_E} \rangle = \rho_E \qquad Var(\frac{E}{N_E}) = \frac{Var(\epsilon)}{N_E} \\
\langle \frac{I}{N_I} \rangle = \rho_I \qquad Var(\frac{I}{N_I}) = \frac{Var(i)}{N_I}
\end{aligned} \tag{16}$$

Exactly at the bifurcation point, the mentioned system size expansion fails because the average of the noise term is unbounded, and therefore, we cannot assume the fluctuating term in equation (4) to be of order $N^{1/2}$. Fig.2 shows how the average and the variance of the membrane potential, the average rates, and the inter-spike interval CV follow the prediction that neurons fire with Poisson statistics, asynchronously and independently. The dashed lines in Fig.2 show the approximation with neurons firing with Poisson statistics and independently with the same rates that we observe in the simulation of the network. The dashed lines in Fig.2C show the rate approximation with the membrane potential distribution being Gaussian with the mean and the variance as predicted in the top panel. Fig.2E shows that the variance and the covariance of the excitatory and the inhibitory rates in the simulation match the values derived from the microscopic model discussed above, i.e., equations (25) and (26) in the main text.

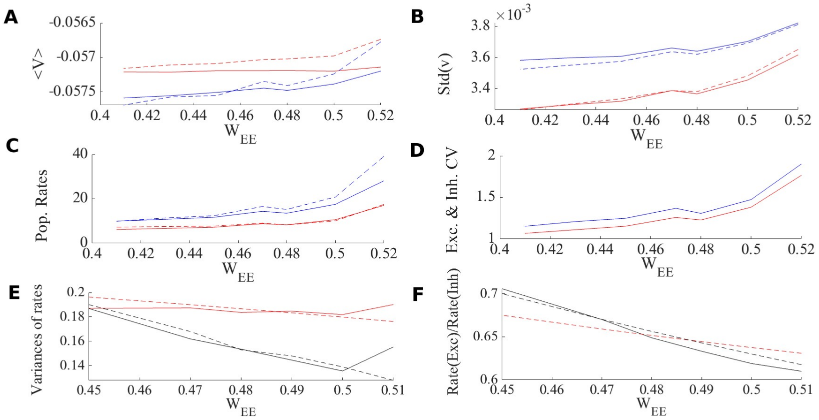

Fig. 2: Characteristics of the network activity for systems with $W_{EI} = 1.5, W_{II} = 2, W_{IE} = 0.75$, $\rho^I_{Ext} = 180Hz, \rho^E_{Ext} = 300Hz$ and $W_{EE} \in [0.41, 52]$ which shows near Poisson firing and avalanche dynamics in smaller values of W_{EE} in the mentioned value range. Red curves show excitatory quantities and blue is for inhibitory ones. Dashed lines are the prediction from the Poisson assumption and solid lines are the simulation results. (A) Average membrane potential. (B) The standard deviation of the membrane potential. (C) Output rates. Here, dashed lines are the firing rates derived from the Gaussian approximation of the potential distribution based on values of the average and the variance of the membrane potential in the top panel of this figure. (D) CV of interspike intervals in the simulation.(E) Ratio of var(E) to var(I) (red) and var(EI) to var(I)(black) in the stationary state of the above mentioned systems. Dashed lines are the approximation derived from the Poisson assumption and solid lines are the simulation results of the spiking neuron network. (F) The ratio of the excitatory to the inhibitory stationary rates varies vs. W_{EE}. The dashed line is $\dfrac{k_{EI}W_{EI}}{k_{EE}W_{EE}}$ and the solid line is the simulation result.

References

Clauset CA. an Shalizi, Newman M (2009) Power-law distributions in empirical data. SIAM Review 51, no 4 (2009): 661–703 http://wwwjstororg/stable/25662336

Van kampen N (2007) Stochastic Processes in Physics and Chemistry. volume in North-Holland Personal Library Book
